# GSNOR Contributes to Demethylation and Expression of Transposable Elements and Stress-Responsive Genes

**DOI:** 10.3390/antiox10071128

**Published:** 2021-07-15

**Authors:** Eva Esther Rudolf, Patrick Hüther, Ignasi Forné, Elisabeth Georgii, Yongtao Han, Rüdiger Hell, Markus Wirtz, Axel Imhof, Claude Becker, Jörg Durner, Christian Lindermayr

**Affiliations:** 1 Institute of Biochemical Plant Pathology, Helmholtz Zentrum München, German Research Center for Environmental Health, 85764 Oberschleißheim, Germany; eva.rudolf@mailbox.org (E.E.R.); elisabeth.georgii@helmholtz-muenchen.de (E.G.); hanyongtao515@163.com (Y.H.); durner@helmholtz-muenchen.de (J.D.); 2 Gregor Mendel Institute of Molecular Plant Biology, Austrian Academy Sciences, Vienna BioCenter (VBC), 1030 Wien, Austria; patrick.huether@gmi.oeaw.ac.at (P.H.); claude.becker@biologie.uni-muenchen.de (C.B.); 3 Genetics, LMU Biocenter, Ludwig-Maximilians-Universität München, 82152 Martinsried, Germany; 4 Protein Analysis Unit, Ludwig-Maximilians-Universität München, 82152 Martinsried, Germany; ignasi.forne@lrz.uni-muenchen.de (I.F.); imhof@lmu.de (A.I.); 5 Centre for Organismal Studies, Ruprecht-Karls-Universität Heidelberg, 69120 Heidelberg, Germany; ruediger.hell@cos.uni-heidelberg.de (R.H.); markus.wirtz@cos.uni-heidelberg.de (M.W.); 6 Chair of Biochemical Plant Pathology, Technische Universität München, 85354 Freising, Germany

**Keywords:** nitric oxide, S-nitrosoglutathione, S-nitrosoglutathione reductase, metaboloepigenetic, S-adenosylhomocysteine, DNA methylation, histone methylation

## Abstract

In the past, reactive nitrogen species (RNS) were supposed to be stress-induced by-products of disturbed metabolism that cause oxidative damage to biomolecules. However, emerging evidence demonstrates a substantial role of RNS as endogenous signals in eukaryotes. In plants, S-nitrosoglutathione (GSNO) is the dominant RNS and serves as the ^•^NO donor for S-nitrosation of diverse effector proteins. Remarkably, the endogenous GSNO level is tightly controlled by S-nitrosoglutathione reductase (GSNOR) that irreversibly inactivates the glutathione-bound NO to ammonium. Exogenous feeding of diverse RNS, including GSNO, affected chromatin accessibility and transcription of stress-related genes, but the triggering function of RNS on these regulatory processes remained elusive. Here, we show that GSNO reductase-deficient plants (*gsnor1-3*) accumulate S-adenosylmethionine (SAM), the principal methyl donor for methylation of DNA and histones. This SAM accumulation triggered a substantial increase in the methylation index (MI = [SAM]/[S-adenosylhomocysteine]), indicating the transmethylation activity and histone methylation status in higher eukaryotes. Indeed, a mass spectrometry-based global histone profiling approach demonstrated a significant global increase in H3K9me2, which was independently verified by immunological detection using a selective antibody. Since H3K9me2-modified regions tightly correlate with methylated DNA regions, we also determined the DNA methylation status of *gsnor1-3* plants by whole-genome bisulfite sequencing. DNA methylation in the CG, CHG, and CHH contexts in *gsnor1-3* was significantly enhanced compared to the wild type. We propose that GSNOR1 activity affects chromatin accessibility by controlling the transmethylation activity (MI) required for maintaining DNA methylation and the level of the repressive chromatin mark H3K9me2.

## 1. Introduction

Nitric oxide (^•^NO) is a ubiquitous signaling molecule with pleiotropic functions throughout the lifespan of plants. Indeed, ^•^NO is involved in the regulation of growth and development processes including seed dormancy [1], seed germination [2,3], root growth [4,5], hypocotyl elongation [6], stomatal closure [7], gravitropism [8], flowering [9,10], pollen tube growth [11], fruit ripening and senescence [12], biotic and abiotic stress responses [13,14,15], and iron homeostasis [16]. In plants, ^•^NO is endogenously produced in different cellular compartments, including the cytosol, peroxisomes, mitochondria, and chloroplasts [17], under both physiological and stress conditions [18].

Although ^•^NO biosynthesis has not been described in the nucleus, it is possibly transferred into the nucleus by passive diffusion, through S-nitrosated proteins or S-nitrosated low-molecular weight thiols, such as S-nitrosoglutathione (GSNO) (discussed in [19]).

^•^NO and reactive nitrogen species (RNS) exert their biological function through post-translational modifications (PTMs), including tyrosine nitration, metal nitrosylation, and S-nitrosation. In general, those ^•^NO-mediated PTMs have profound effects on the function of target proteins by regulating their activities, subcellular localization, structure, or interaction with biomolecules [20,21,22]. Protein S-nitrosation is the most important ^•^NO-mediated PTM [14]. Proteome-wide studies identified putatively S-nitrosated proteins involved in numerous aspects of plant biology, such as plant immune response, the antioxidant system, metabolic processes, and transcription factors. Consequently, ^•^NO regulates diverse physiological processes by altering gene expression [23,24,25], metabolite levels [26], and/or phytohormone signaling [27,28].

^•^NO is a short-lived free-radical, whose function is restricted to its local microenvironment. In contrast, GSNO is a more stable redox form of ^•^NO [29,30] regarded as an intracellular mobile ^•^NO reservoir [31], which can release ^•^NO in the presence of metal ions, such as copper and iron, or reductants, such as ascorbate or GSH [32]. Moreover, GSNO can transfer its ^•^NO moiety directly to cysteine thiol groups of other proteins (trans-nitrosation) [22,29]. GSNO levels are controlled by the activity of S-nitrosoglutathione reductase (GSNOR). GSNOR is an evolutionarily conserved enzyme that catalyzes the NADH-dependent reduction of GSNO to oxidized GSH (GSSG) and ammonia in the presence of GSH [29,30]. The *Arabidopsis* genome encodes a single-copy *GSNOR* gene. Loss of GSNOR1 leads to elevated levels of ^•^NO, nitrate, nitrite, and S-nitrosothiols (RSNOs), and a proteome-wide increased S-nitrosation [33,34,35,36,37,38,39]. Hence, GSNOR1 is considered to control the intracellular levels of both GSNO and, indirectly, protein SNOs [34]. GSNOR1 deficiency causes pleiotropic plant growth and development defects, impaired plant disease responses, heat sensitivity, and resistance to cell death [29,30,31], suggesting a regulatory role of GSNOR in these processes [29,30,31].

A number of nuclear proteins that undergo S-nitrosation have been identified [40], suggesting a regulatory function of GSNO/^•^NO in nuclear events/processes. Apart from the transcriptional or post-translational control of transcription factors [41], several lines of evidence demonstrate that GSNO/^•^NO regulates gene expression also via modulation of the chromatin structure and/or DNA accessibility [23]. In general, the distinct chromatin states that modulate access to DNA for transcription are regulated by multiple epigenetic mechanisms, including DNA methylation, covalent modifications of core histones such as methylation and acetylation, ATP-dependent chromatin remodeling, placement of histone variants, non-coding RNAs, and metaboloepigenetic effects [42,43]. In *Arabidopsis*, GSNO treatment induced histone hyperacetylation at genes related to stress response by inhibiting histone deacetylase activity [44]. Further, the plant-specific histone deacetylases HDT1/2 regulating the expression of *GIBBERELLIN 2-OXIDASE2* by histone acetylation [45] were identified as targets for S-nitrosation [40]. DNA hypomethylation concomitant with transcriptional activation of transposable elements (TEs) was observed in rice upon treatment with 0.1–1 mM of the ^•^NO donor sodium nitroprusside [46].

From bacteria to humans, methylation is sensitive to the cellular metabolic status [47]. Both the methylation cycle and the tricarboxylic acid cycle provide substrates for enzymes involved in DNA and histone methylation. Indeed, methylation is directly linked to intermediary metabolism with S-adenosylmethionine (SAM) acting as the main methyl donor for transmethylation reactions catalyzed by methyltransferases, which methylate DNA, RNA, lipids, histones, and cellular metabolites [48]. Each transmethylation reaction consumes SAM and releases the by-product S-adenosylhomocysteine (SAH). SAH, a competitive inhibitor of methyltransferases, is then recycled to homocysteine (Hcys) and adenosine by S-adenosylhomocysteine hydrolase (SAHH). The equilibrium of this reversible reaction favoring SAH is driven towards hydrolysis of SAH due to removal of its products by downstream enzymes. Methionine synthase (MS) catalyzes the methylation of Hcys to methionine using methyl-tetrahydrofolate (CH_3_-THF). Then, S-adenosylmethionine synthetase (SAMS) catalyzes the adenylation of methionine to SAM to close the methylation cycle [48,49]. The recycling mechanism is crucial for maintaining an adequate methylation index (MI; SAM/SAH ratio), which is regarded as an indicator of the cellular methylation state.

Numerous studies reported that SAM and SAH levels regulate DNA and histone methylation [42,50,51]. The *Arabidopsis* genome encodes two *SAHH* isoforms; however, *SAHH*1 is assumed to play a predominant role in maintaining TGS and DNA methylation at numerous targets compared to *SAHH2* [52,53]. *Arabidopsis sahh1* knock-down mutants (*sahh1-kd;* knockout is zygotic lethal [52]) possessed a decreased MI [52,54], as well as decreased DNA and H3K9me2 methylation, concomitant with the release of transcriptional silencing at transgene reporters [52,53], repetitive DNA sequences such as ribosomal DNA and 180 bps repeats [52,53,54], and transposons [55,56]. Similarly, the expression of antisense RNA of *SAHH* in tobacco plants resulted in a loss of DNA methylation in repetitive elements [57]. Other studies employed a selective reversible inhibitor of SAHH, namely, dihydroxypropyladenine (DHPA). In tobacco, DHPA caused accumulation of SAH and DNA hypomethylation [58,59,60]. In *Arabidopsis*, the application of DHPA reduced levels of DNA and histone methylation at endogenous repeats [53]. Moreover, SAMS4 is an important epigenetic regulator in *Arabidopsis*. Mutations in *SAMS4* caused decreased SAM levels, CHG/CHH and H3K9me2 hypomethylation, and activation of TEs [61]. Similarly, *MS1* mutation resulted in a decreased MI, and decreased DNA and H3K9me2 hypomethylation [50]. Accordingly, overexpression of *MS1* is accompanied by a genome-wide global increase in DNA methylation in *Arabidopsis* [62].

Here, we report that the GSNOR1 function is required for SAM homeostasis, and, hence, for balancing the methylation index (ratio of SAM/SAH). Consequently, loss of GSNOR1 activity affects transmethylation reactions. Nano-liquid chromatography mass spectrometry (LC-MS) profiling of histone modifications demonstrated a significant global increase in the repressive H3K9me2 mark in *gsnor1-3*. Whole-genome bisulfite sequencing and transcriptome analyses revealed enhanced DNA methylation and reduced expression of TEs and stress-responsive genes in *gsnor1-3*, in comparison to the wild type. Our data suggest that the GSNOR1 function is required to reduce the level of the repressive chromatin mark H3K9me2, which is associated with the silencing of repeats and TEs. This function might be link to the activation of stress response genes.

## 2. Materials and Methods

### 2.1. Plant Material and Cultivation

*A. thaliana* ecotype Columbia-0 (Col-0; wt) purchased from the Nottingham Arabidopsis Stock Center (NASC), *gsnor1-3* obtained from GABI-Kat (also named *hot5-2*, GABI-Kat 315D11), *sahh1* purchased from NASC (SALK 068487), and the *A. thaliana* Col-0 *TS-GUS* (possesses a transcriptionally silent (TS), highly repetitive β-glucuronidase (GUS) transgene; L5, 6b5) line kindly provided by Hervé Vaucheret were used in this study and were previously described [34,35,53,54,56,63,64]. The *A. thaliana* Col-0 *TS-GUS* (L5, 6b5) line [64] was crossed with the mutants *sahh1* and *gsnor1-3*. The segregating F2 plants were genotyped, and seeds from lines homozygous for the *TS-GUS* locus and the mutation were used for further analysis. Oligonucleotides are listed in Supplemental Table S1.

*Arabidopsis* plants were grown on soil mixed with silica sand in a ratio of 4:1 in 4-well plant pots placed in a tray. Before sowing, soil was wetted with water supplemented with 0.15% (*v/v*) Neudorff Neudomück^®^. After stratification for two days at 4 °C in the dark, plants were cultivated for four weeks in a climate chamber at 65–68% relative humidity under long-day conditions (14 h light/10 h dark cycle, 20 °C day/18 °C night regime, 70 µmol m^−2^ s^−1^ photon flux density). Four-week-old rosette leaves were harvested 5 h after the day-time start and flash frozen in liquid nitrogen.

For liquid culture experiments, *A. thaliana* seeds were surface sterilized by soaking in 70% (*v/v*) ethanol for 1 min and then in 50% (*v/v*) household bleach for 10 min followed by five washes with sterile ddH_2_O. Seeds were suspended in sterile water and stratified for 2 days at 4 °C in the dark. Seedlings were cultivated in six-well plates containing 5 mL of 1x Murashige and Skoog (MS) medium [65] adjusted to pH 5.7 with potassium hydroxide and supplemented with 1% sucrose and 0.5 g L^−1^ MES. Plantlets were germinated and grown for twelve days in liquid media supplemented with 200 µM DHPA or water (mock) under short-day conditions (10 h light/14 h dark cycle, 16 °C day/ 20 °C night regime, relative humidity 80% day/65% night, 100 µmol m^−2^ s^−1^ photon flux density) on a shaker (100 rpm). Media including drugs were exchanged every day at the night-time start. 

### 2.2. Quantification of MTA, SAM, SAH, Cys GSH, and Hcys 

Samples (0.1 g) were ground in liquid nitrogen to a fine powder and extracted with 0.1 M HCl (0.1 mL) by vortexing at 4 °C for 15 min. The resulting homogenates were centrifuged for 10 min at 4 °C and 16,400 *g* to remove cell debris. Adenosines were derivatized with chloro-acetaldehyde as previously described [66]. The metabolites were separated by reversed-phase chromatography on an Acquity HSS T3 column (100 mm × 2.1 mm, 1.7 µm, Waters) equilibrated in buffer A (5.7 mM TBAS, 30.5 mM KH2PO4 pH 5.8) by applying the following gradient: 0.6 mL 1% B, 1.9 mL 8% B, 1.9 mL 14% B, 5.7 mL 50% B. Buffer B was a mix of 34% buffer A and 66% acetonitrile. The fluorescent 1,N^6^-etheno-derivatives of MTA, SAM, and SAH were quantified with an Acquity FLR detector (Waters, excitation: 280 nm, emission: 410 nm) connected to an H-class UPLC system. The thiols (Cys, GSH, and Hcys) were labeled with monobromobimane, and the resulting fluorescent thiol-bimane derivates were separated by reversed-phase chromatography according to (Dong et al., 2017).

### 2.3. Whole-Genome Bisulfite Sequencing and Data Analysis

*WGBS library preparation and sequencing.* WGBS was performed from snap-frozen 4-week-old rosette leaves grown under long-day conditions and harvested 5 h after the day-time start (total 1.5 g) for each genotype. Two biological replicates were analyzed for each genotype. gDNA was extracted from leaf samples (aliquot 150 mg, ground in liquid nitrogen) with DNeasy^®^ Plant Mini Kit and sheared to 350 bps. WGBS DNA libraries were generated using the Illumina^®^ TruSeq^®^ Nano Kit, and bisulfite treatment was conducted with the EpiTect^®^ Plus Bisulfite Kit. Briefly, the fragmented DNAs were end repaired, adenine bases were added to the 3’end (A-tailing) of the DNA fragments, and methylated adapters were ligated to the DNA fragment. Next, the DNA fragments were size selected before sodium bisulfite treatment and PCR amplification (KAPA HiFi HS Uracil+ R from Roche Cat.No:795905001). Libraries were sequenced with 125 bp paired-end reads on a Hiseq 2500 instrument.

*Processing and alignment of bisulfite-converted reads.* For read mapping, the nf-core methylseq pipeline available at https://github.com/nf-core/methylseq (accessed on 12 February 2018 (https://doi.org/10.5281/zenodo.1343417; accessed on 9 May 2021) was used. In short, raw sequencing reads were quality controlled (FastQC v0.11.5), and sequencing adapters were trimmed off (Trim Galore v0.4.1). Reads were aligned to the TAIR9 Reference genome with Bismark (version v0.17.0) [67] using the Bowtie2 aligner [68]. After deduplication (picardtool MarkDuplicates v2.8.0), methylated Cs were extracted from aligned reads with MethylExtract (v1.9.1). Bisulfite conversion efficiency was calculated from the proportion of unconverted Cs in the chloroplast genome.

*Post-alignment Analysis.* Methylation calling information of each individual cytosine was tabulated and subjected to post-alignment analysis with the MethylScore pipeline. Briefly, identification of differentially methylated positions was performed according to Becker et al. [69]. Identification of methylated regions (MRs) and differentially methylated regions (DMRs) was conducted by an adaption of a hidden Markov model-based approach, as previously described [70], which identifies regions of dense methylation that are then tested for differential methylation [71]. The DMRs were identified by pairwise comparison of WGBS profiles (*gsnor1-3* vs. Col-0/wt; *sahh1* vs. Col-0/wt). 

*Annotation—mapping to genomic elements.* For annotation of genomic elements, the TAIR10 reference annotation was used. MRs and DMRs were assigned to annotated elements (CDS, intron, 5′ UTR, 3′ UTR, transposon, 2kb upstream, 2kb downstream, as-lncRNA, lncRNA, miRNA, pri-miRNA, ncRNA, snoRNA, tRNA, pseudogene). Genes with at least one DMR in the gene body, at 3kb up- or downstream of flanking regions, were considered as differentially methylated genes (DMGs). Further, TEs with at least one DMR were identified.

### 2.4. RNA Sequencing

RNA-seq was performed from snap-frozen 4-week-old rosette leaves grown under long-day conditions and harvested 5 h after the day-time start (total 1.5 g) for each genotype. Four replicates were analyzed for each genotype. RNA was extracted from 4-week-old rosette leaves using the innuPREP PLANT RNA Kit. Sequencing libraries were generated from Poly(A)-enriched RNA using the NEBNext^®^ Ultra^™^ II Directional RNA Library Prep kit (New England Biolabs) according to the manufacturer’s instructions and sequenced on a HiSeqV4 instrument (Illumina) as 100 bp single-end reads. Reads were mapped to the TAIR10 reference of *Arabidopsis thaliana* annotated genes (www.arabidopsis.org; accessed on 24 December 2019) using STAR (v2.5.2a) [72]. Read quantifications were generated using Kallisto (v0.43.1) [73]. Differential expression analysis was performed using the DESeq2 package (v1.18.1) in R [74]. Gene annotation was performed using the following sources: UniProtKB, Swiss-Prot, TrEMBL, and TAIR. 

### 2.5. Acid Extraction of Histones

Nuclei from 4-week-old rosette leaves were purified as described previously [75], with minor modifications. Two grams of plant tissue was grinded to a fine powder in liquid nitrogen, homogenized in two volumes of lysis buffer (20 mM Tris-HCl pH 7.4, 25% (*v/v*) glycerol, 20 mM KCl, 2 mM EDTA, 2.5 mM MgCl_2_, 250 mM sucrose) supplemented with protease inhibitor, and incubated for 10 min on ice with intermittent vortexing. The homogenate was successively filtered through miracloth and a 160 µm nylon mesh. The flow-through was centrifuged at 1500 *g* for 10 min at 4 °C, and the pellet was washed four times with 4 mL of nuclear resuspension buffer (20 mM Tris-HCl pH 7.4, 25% (*v/v*) glycerol, 2.5 mM MgCl_2_, 0.2% (*v/v*) Triton^®^ X-100). The final pellet was resuspended in 700 µL of 0.2 M sulfuric acid to extract histones and other acid-soluble proteins overnight using an overhead shaker. The extract was then centrifuged at 16,000× *g* for 20 min at 4 °C. The supernatant containing core histones was transferred to a new tube, and proteins were precipitated with 26% (*v/v*) trichloroacetic acid for 3 h on ice. After four washes with ice-cold acetone, the histone pellet was resuspended in 60 µL of 2× sample buffer (4% (*w/v*) SDS, 20% (*v/v*) glycerol, 10% (*v/v*) 2-mercaptoethanol, 0.004% (*w/v*) bromo-phenol blue, and 0.125 M Tris-HCl, pH 6.8) at 1500 rpm for 20 min at RT using a thermoshaker and then stored at −20 °C overnight. If necessary, pH was adjusted with 1 µL of 1 M Tris-HCl pH 8.0. Histones were separated by 12% SDS-PAGE (loading volume: 15–25 µL per lane) and stained with Coomassie^®^ Blue for LC-MS/MS analysis or immunoblotted.

### 2.6. Quantification of Histone Methylation and Acetylation by LC-MS/MS

After electrophoreses, bands corresponding to histones H3 and H4 were excised from the gels. Calf histones were run as a size marker. Destaining, d3-acylation, digestion, and peptide desalting were performed as described before [76], with minor modifications. Desalted histone peptides in 0.1% TFA were injected in an RSLCnano system (Thermo Fisher Scientific) and separated in a 15 cm analytical column (75μm ID home-packed with ReproSil-Pur C18-AQ 2.4 μm from Dr. Maisch), with a 50 min gradient from 4 to 40% acetonitrile in 0.1% formic acid at a 300 nL/min flowrate. The effluent from the HPLC was electrosprayed into a Q Exactive HF mass spectrometer (Thermo Fisher Scientific). The MS instrument was programmed to target several ions as previously described [76], except for the MS3 fragmentation. Survey full-scan MS spectra (from m/z 270–730) were acquired with resolution R = 60,000 at m/z 400 (AGC target of 3 × 10^6^). Targeted ions were isolated with an isolation window of 0.7 m/z to a target value of 2 × 10^5^ and fragmented at 27% normalized collision energy. Typical mass spectrometric conditions were: spray voltage, 1.5 kV; no sheath and auxiliary gas flow; heated capillary temperature, 250 °C.

Peptide and fragment masses of histone H3 methylation and acetylation marks were calculated in GPMAW [77]. The histone PTMs were quantified based on the area of the peak from the extracted ion chromatogram, and the Xcalibur™ software v2.2 SP1 (Quan Browser, Thermo Fisher Scientific) was used. The theoretical mass to charge ratio was calculated with GPMAW 5.02 for each peptide. Further parameters were peak detection: Genesis; trace: mass range; mass tolerance: 20 ppm; mass precision: 4 decimals; S/N threshold: 0.5. After peak integration, data were exported to Excel, and the relative abundance was calculated as previously described [76].

### 2.7. Accession Numbers

Assigned accession numbers for the genes used in this work from The Arabidopsis Information Resource (www.arabidopsis.org accessed on 24 December 2019) are as follows: AT4G13940 (*SAHH1*), AT5G43940 (*GSNOR1*). RNA-seq and WGBS data were stored in the European Nucleotide Archive (ENA) under the ENA accession number PRJEB43942. 

## 3. Results 

### 3.1. GSNOR and SAHH1 Are Involved in Regulating Metabolite Levels of the Methylation Cycle

The function of GSNOR bioactivity in regulating metabolite levels of the methylation cycle was investigated in vivo using a GSNOR-deficient line, namely, *gsnor1-3* [34,35]. The GSNOR1-deficient mutant (*gsnor1-3*; also named *hot5-2*) is an important tool for functional analysis of GSNO under physiological conditions. Since in this mutant, the enzymatic degradation of GSNO is abolished, the observed phenotypical and molecular effects directly reflect the functions of SNOs in vivo [29,31]. Besides the Col-0 wild type (wt), the *sahh1* knock-down mutant [54,56,63] was used as a control plant (Supplemental Figure S1A). Both, GSNOR activity and the total RSNO content were analyzed in *gsnor1-3* and *sahh1* mutants under basal conditions. GSNOR activity significantly decreased to 10% in *gsnor1-3* relative to wt plants (Supplemental Figure S1B). The decreased GSNOR activity in the *gsnor1-3* mutant was accompanied by an almost 2-fold increase in RSNO levels (Supplemental Figure S1C). These results are in accordance with previous studies [34,35,39]. Neither the GSNOR activity nor the RSNO content is significantly different between *sahh1* and wt (Supplemental Figure S1B,C). 

**Figure 1:**
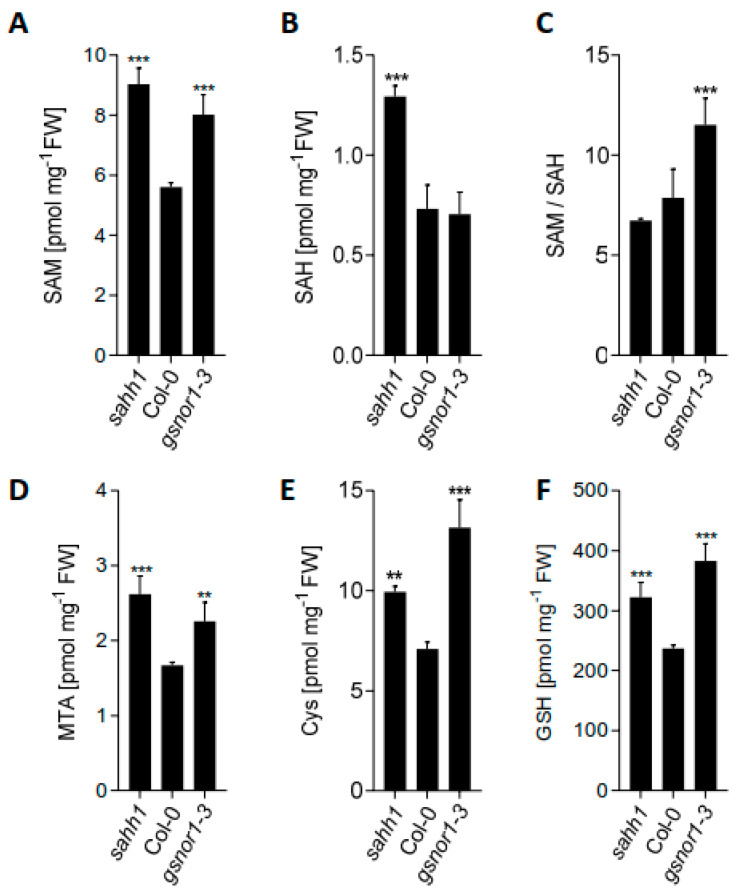
**Mutations in *GSNOR1* and S*AHH1* result in an impaired methylation cycle.** Analysis of steady-state levels of (**A**) SAM, (**B**) SAH, (**C**) SAM/SAH, (**D**) MTA, (**E**) Cys, and (**F**) GSH in 4-week-old rosette leaves grown under long-day conditions and harvested 5 h after the day-time start (*n* = 5). Values are normalized against total fresh weight and represent the mean ± SD. Grubb´s outlier test (α = 0.05) was performed. ** (*p* < 0.01) and *** (*p* < 0.001) represent significant differences between wt and mutants (ANOVA with Dunnett´s multiple comparisons test). Statistical analysis was performed with GraphPad Prism version 7.05.

To analyze whether GSNOR1 and SAHH1 functions are required for intact processing of the methylation cycle and connected pathways, we determined the steady-state levels of SAM, SAH, and Hcys (methylation cycle), cysteine (Cys) and glutathione (GSH) (glutathione biosynthesis), and 5´-methylthioadenosin (MTA; by-product of polyamine, ethylene, and nicotianamine biosynthesis) in wt, *sahh1*, and *gsnor1-3* (Figure 1A–B,D–F). Moreover, we calculated the SAM/SAH ratio, also named the methylation index (MI), which is regarded as an indicator of the cellular methylation state (Figure 1C). The major methyl group donor SAM was significantly elevated in *sahh1* and *gsnor1-3* plants by 61% and 43%, respectively (Figure 1A). The *sahh1* mutant also showed a 2-fold increase in the SAH level, resulting in an overall decrease in the MI by 14% compared to wt (Figure 1B,C). This result is concordant with previous studies [52,54]. Since the SAH level of *gsnor1-3* was similar to that of wt, the resulting SAM/SAH ratio was significantly increased by 47% (Figure 1C). Hcys levels were below the detection limit in all three lines (data not shown), whereas the levels of MTA, Cys, and GSH were significantly increased in *gsnor1-3* and *sahh1* in comparison to wt (Figure 1D–F). In conclusion, target metabolomic analysis in wt, *sahh1*, and *gsnor1-3* revealed alterations in the levels of SAM, SAH, MTA, Cys, and GSH, acting as precursors of substrates, cofactors, or inhibitors in epigenetic methylation processes. These results suggest that SAHH1 and GSNOR1 functions might be linked to histone and DNA methylation. 

Interestingly, SAHH1 was identified as S-nitrosated under basal and stress conditions in proteome-wide studies [33,78,79,80,81] and in *gsnor1-3* seedlings [33], and several groups demonstrated that tyrosine nitration and S-nitrosation strongly inhibit SAHH1 activity in vitro [82]. We confirmed that recombinant SAHH1 can be S-nitrosated and reversibly inhibited by GSNO (Supplemental Figure S2A,B). SAHH1 is also inhibited by the sulfhydryl-modifying agent N-ethylmaleinimide (NEM), confirming that cysteine residues are important for its activity (Supplemental Figure S2B). However, although *gsnor1-3* has an enhanced level of RSNOs, in vivo S-nitrosation of SAHH1 could not be detected (Supplemental Figure S2C). This, together with the fact that SAH (and Hcys) levels are unchanged in *gsnor1-3*, in comparison to wt, suggests that loss of the GSNOR function is not linked to inhibition of SAHH1 under the analyzed conditions.

### 3.2. Loss of GSNOR1 and SAHH1 Functions Results in Altered Histone Methylation Levels

To investigate the consequence of the altered metabolite levels and the MI in *gsnor1-3* and *sahh1* on histone modification, 4-week-old rosette leaves were analyzed by LC-MS [76]. The analysis of histone H3 revealed that the H3K9me2 level significantly increased by 23% and significantly decreased by 34% in *gsnor1-3* and *sahh1*, respectively, relative to wt (Table 1). 

**Figure 2:**
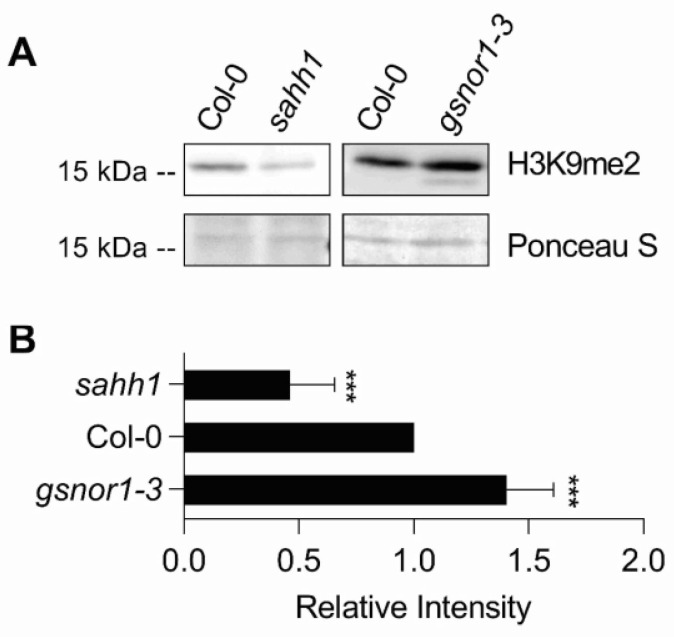
**Histone H3K9me2 methylation level is altered in *gsnor1-3* and *sahh1***. (**A**) H3K9me2 immunoblot. Histones were acid extracted from 4-week-old rosette leaves grown under long-day conditions and harvested 5 h after the day-time start and probed against H3K9me2 marks by immunoblotting. As the loading control, the Ponceau S-stained membrane is shown. One representative experiment is shown. (**B**) Quantification of immunoblot results. Signal intensities were measured using ImageJ software and normalized to the amount of loaded H3. Statistics: values are expressed as fold change over wt and represent the mean ± SD of at least three independent experiments (*n* = 4–7). Grubb´s outlier test (α = 0.05) was performed. *** (*p* < 0.001) represents significant differences between wt and mutant lines (ANOVA, Dunnett´s multiple comparisons test). Statistical analysis was performed with GraphPad Prism version 7.05.

The altered H3K9me2 levels were confirmed by immunoblotting using an anti-H3K9me2 antibody (Figure 2 A,B). In addition, the H3.1K27me2 mark was significantly increased by 23% in *gsnor1-3* plants. 

### 3.3. SAHH1 and GSNOR1 Functions Affect DNA Methylation

Since H3K9me2 is functionally linked to DNA methylation [43,83,84], we postulated that the observed altered global H3K9me2 level in *sahh1* and *gsnor1-3* plants would entail changes in DNA methylation. 

**Figure 3:**
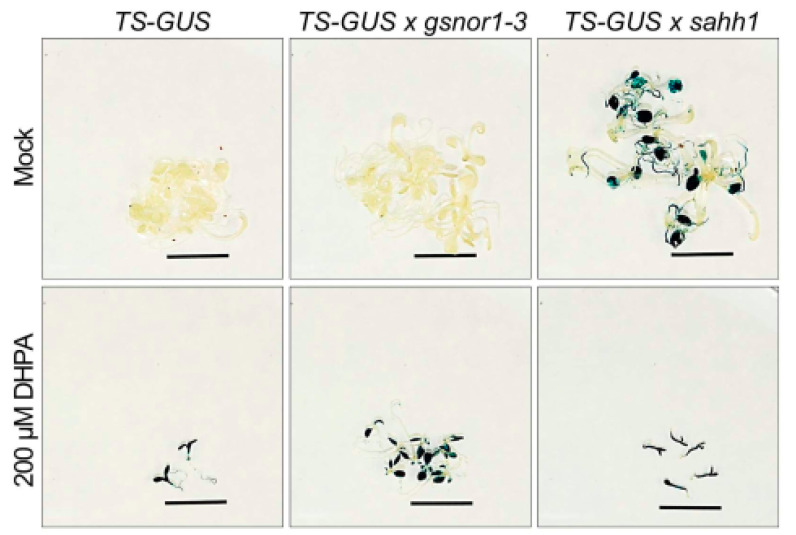
**Reactivation of *TS-GUS* in *gsnor1-3* and *sahh1*.** Blue staining indicates release of gene silencing. Plantlets were grown in liquid 1× MS under short-day conditions supplemented with water (mock) as control, or seedlings were grown in the presence of 200 µM DHPA. Due to DHPA, reduced growth is observed as previously demonstrated [53]. GUS reactivation was visible in *sahh1* but not in the *gsnor1-3* background. Treatments with the SAHH inhibitor DHPA released TS-GUS silencing in all mutant backgrounds. Scale bar = 1 cm.

We used the *A. thaliana* Col-0 *TS-GUS* (L5, 6b5) line, which possesses a transcriptionally silent highly repetitive GUS transgene on chromosome III [64], to analyze the effect of GSNOR1 and SAHH1 on DNA methylation. Transcriptional gene silencing (TGS) is generally concomitant with high levels of DNA methylation and inactive chromatin marks such as H3K9me2. We crossed the *TS-GUS* (L5) line with *sahh1* and *gsnor1-3* mutant lines (Supplemental Figure S3) and assessed the reactivation of TS-GUS in 10-day-old seedlings (Figure 3). As a control, seedlings were grown in the presence of DHPA, an SAHH-specific inhibitor previously demonstrated to reactivate *TS-GUS* [53]. DHPA induced the reactivation of the GUS transgene in each mutant background. However, in the absence of DHPA, activation of the GUS transgene was only observed in the *sahh1* background, but not in the *gsnor1-3* background. These results demonstrate a hypomethylation phenotype of *sahh1* and argue for an unchanged methylation status or a hypermethylation phenotype of *gsnor1-3*.

DNA methylation in *sahh1* and *gsnor1-3* was further analyzed by whole-genome bisulfite sequencing (WGBS). WGBS allows studying genome-wide DNA methylation at single-nucleotide resolution. To assess the bisulfite conversion efficiency, reads were mapped to the non-methylated chloroplast genome, resulting in an average conversion rate of more than 98%. The mean methylation levels per DNA methylation context (CG, CHG, or CHH, where H = A, C, or T) are listed in Table 2. 

**Figure 4:**
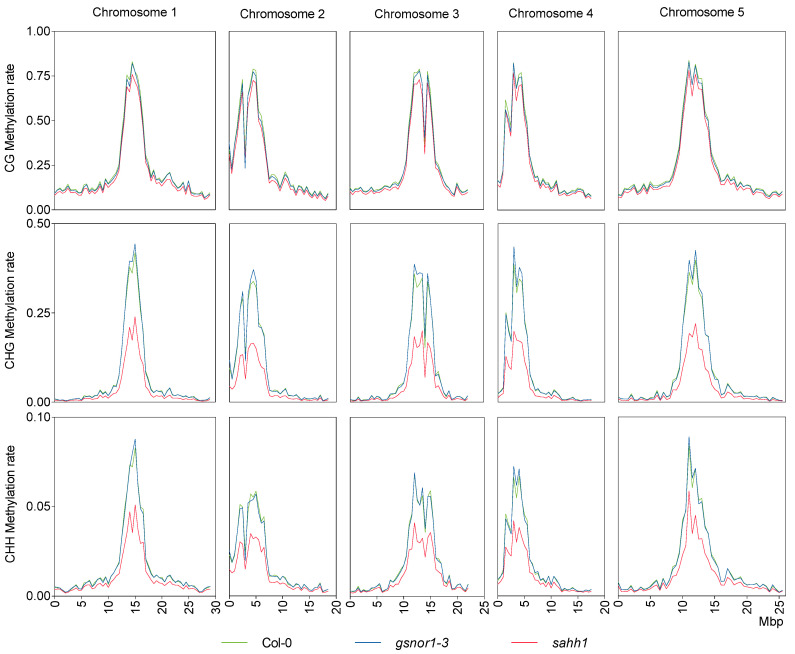
**Chromosomal distribution of DNA methylation is altered in *gsnor1-3* and *sahh1.*** The methylation levels across the chromosomes in each sequence context were calculated with MethGeno [86] for each replicate. Then, replicates were merged, and graphs were made with GraphPad Prism. Average methylation of all cytosines within a 0.5 Mbp interval is plotted.

These data are in accordance with the average methylation levels of 24% CG, 7% CHG, and 2% CHH found in *Arabidopsis* [85]. The mean methylation rates in *gsnor1-3* are similar to those in wt, whereas *sahh1* shows decreased methylation rates (Table 2). However, at the level of chromosomal distribution, hypermethylation in *gsnor1-3* was most pronounced in the TE-rich pericentromeric regions in the CHG context (Figure 4). For *sahh1*, we observed the strongest effect in the CHG context, followed by CHH and CG compared to wt methylation rates (Table 2). Loss of methylation in *sahh1* was unevenly distributed along the chromosomes and was most pronounced in the highly methylated TE-rich pericentromeric regions, particularly for CHG and CHH (Figure 4). Taken together, DNA methylation is altered in both mutants compared to wt.

### 3.4. GSNOR1 and SAHH1 Regulate DNA Methylation of TEs and Genes

**Figure 5:**
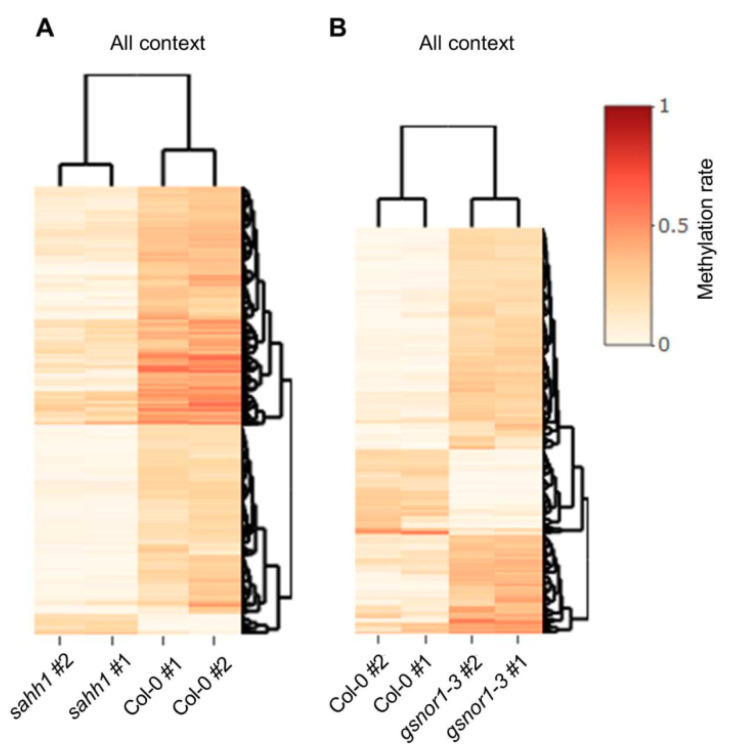
**Enrichment of hypo- and hyper-DMRs in *sahh1* and *gsnor1-3*, respectively.** Heatmaps of hierarchically clustered DMRs identified in pairwise comparisons of wt vs. *sahh1* methylome (**A**) and wt vs. *gsnor1-3* methylome (**B**). DNA was extracted from 4-week-old rosette leaves grown under long-day conditions, harvested 5 h after the day-time start, and subjected to WGBS. Heatmaps represent the methylation level across DMRs: red = 100% methylated, white = 0% methylated. Two biological replicates were analyzed for each genotype.

To assess whether GSNOR1 and SAHH1 affect the methylation status of the defined genomic regions, we first called methylation regions (MRs) using the adaptation of a two-state hidden Markov model-based approach and identified differentially methylated regions (DMRs) in pairwise comparisons (*gsnor1-3* vs. wt, and *sahh1* vs. wt) according to Hagmann et al. [70]. We identified 42,304 and 40,305 MRs in wt and *gsnor1-3*, respectively. Comparing wt and *sahh1* resulted in 42,288 and 51,223 identified MRs, respectively. DMR identification in pairwise comparisons (mutant vs. wt) revealed 752 and 292 DMRs for *sahh1* and *gsnor1-3* (Supplemental Table S2), respectively. In *sahh1*, 35 DMRs were hypermethylated and 717 were hypomethylated relative to wt (Figure 5A), in line with the overall decrease in *sahh1* (Table 2). In contrast, *gsnor1-3* showed considerably more hypermethylated (231) than hypomethylated (61) DMRs relative to wt (Figure 5B), despite the fact that mean methylation rates were similar to those of wt (Table 2). In summary, *sahh1* and *gsnor1-3* mutants predominantly showed local hypo- and hypermethylation, respectively. 

Genomic feature annotation showed that DMRs mainly mapped to the genic (CDS), 3kb up- or downstream flanking regions of genes (hereafter, genes overlapping with identified DMRs in their genic, 3kb up- and/or downstream region are summarized as differentially methylated genes (DMGs)) and TEs (Supplementary Figure S4A,B).

**Figure 6:**
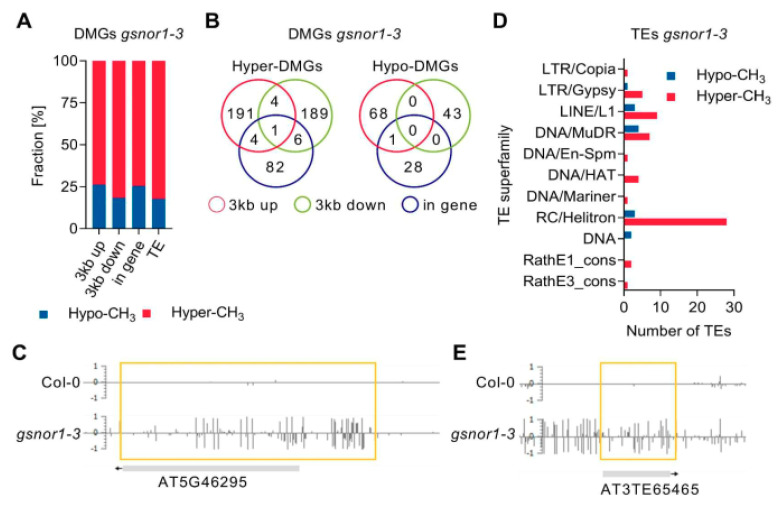
**Loss of GSNOR1 function results in an enrichment of hypermethylated DMGs and TEs.** (**A**) Stacked bar plot showing the fraction of hyper- and hypo-DMGs and TEs with respected to DMRs found in their genic, 3kb up- or downstream region or TE coding region. (**B**) Venn diagram summarizing DMGs with DMRs in multiple genomic features (genic, 3kb up- and/or downstream flanking regions). (**C**) Snapshot of AT5G46295 in the EPIC-CoGE browser. (**D**) Distribution of differentially methylated TEs over TE superfamilies. (**E**) Snapshot of AT3TE65465 in the EPIC-CoGE browser. DNA methylation data have been uploaded to the epigenome browser of EPIC (EPIC-CoGE) by Prof. Dr. Claude Becker (ID 2234 unpublished). DNA methylation analysis was performed in duplicates, and average methylation ratios calculated in the CoGE browser are shown. TE classification according to www.arabidopsis.org accessed on 24 December 2019.

In detail, loss of the GSNOR1 function resulted in an enrichment of hypermethylated DMGs and TEs (Figure 6A). In total, 587 DMGs were identified in *gsnor1-3* (Supplemental Table S3). Among those, 449 were hypermethylated and 138 were hypomethylated. DMGs with DMRs in multiple genomic elements were identified, as illustrated in the Venn diagram (Figure 6B). For instance, hypermethylation of AT5G46295 encoding a transmembrane protein is observed in its 3kb upstream flanking and genic region (Figure 6C). Loss of the GSNOR1 function resulted in an enrichment of hypermethylated TEs (Figure 6A,D). In detail, 55 and 12 TEs overlapping with hyper- and hypomethylated DMRs were identified (Supplemental Table S4), respectively. TEs classified as retrotransposons in the superfamilies LTR/Gypsy and LINE/L1, as well TEs classified as DNA transposons belonging to the superfamilies MuDR and RC/Helitron, were mainly hypermethylated in *gsnor1-3* (Figure 6D). A snapshot in the EPIC-CoGE browser of a representative TE (AT3TE65465, LTR/Gypsy) overlapping with a hyper-DMR is shown in Figure 6E.

**Figure 7:**
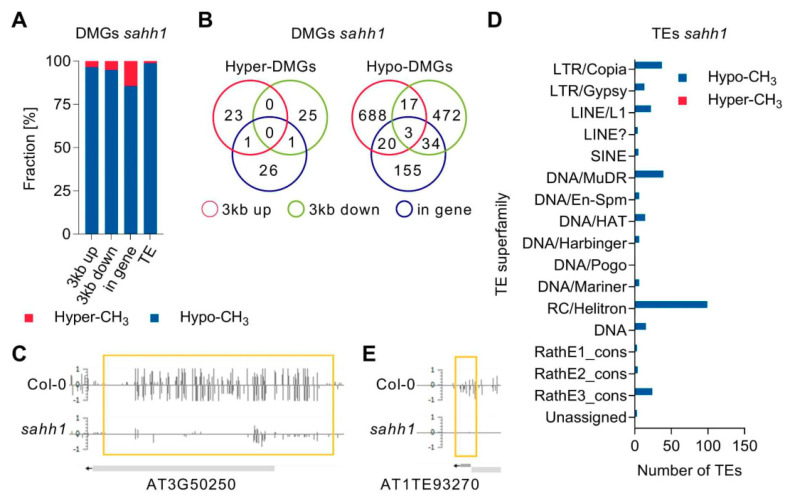
**Knock-down of SAHH1 function results in an enrichment of hypomethylated DMGs and TEs.** (**A**) Stacked bar plot showing the fraction of hyper- and hypo-DMGs and TEs with respect to DMRs found in their genic, 3kb up- or downstream region or TE coding region. (**B**) Venn diagram summarizing DMGs with DMRs in multiple genomic features (genic, 3kb up- and/or downstream flanking regions). (**C**) Snapshot of AT3G50250 in the EPIC-CoGE browser. (**D**) Distribution of differentially methylated TEs over TE superfamilies. (**E**) Snapshot of AT1TE93270 in the EPIC-CoGE browser. DNA methylation analyses were performed in duplicates, and average methylation ratios calculated in the CoGE browser are shown. TE classification according to www.arabidopsis.org (24 December 2018).

*SAHH1* knock-down resulted mainly in an enrichment of hypomethylated DMGs and TEs (Figure 7A). In *sahh1* plants, 1299 DMGs were identified (Supplemental Table S5). Among those, 72 are hypermethylated and 1227 are hypomethylated. Of note, three of those DMGs in *sahh1* possess hyper- and hypo-DMRs (At1g65220, At3g54730, At4g13440). As illustrate in the Venn diagrams, DMGs with DMRs in multiple genomic elements were identified (Figure 7B). For instance, AT3G50250 encoding a transmembrane protein is hypomethylated in its 3kb upstream flanking and genic region (Figure 7C). 

TEs were mainly hypomethylated in *sahh1* (Figure 7A,D; Supplemental Table S6). In detail, 3 TEs and 271 TEs with hyper- and hypomethylated DMRs were identified, respectively. Hypomethylation was mainly found in members of the retrotransposon superfamilies LTR/Copia and LINE/L1, and in members of the DNA transposon superfamilies MuDR and RC/Helitron (Figure 7D). A snapshot in the EPIC-CoGE browser of a representative hypomethylated TE (AT1TE93270, DNA/HAT) is shown in Figure 7E. Gene Ontology enrichment analysis of DMGs identified in *gsnor1-3* and *sahh1* did not result in significantly enriched GO terms.

### 3.5. Transcriptomic Profiling of gsnor1-3 and sahh1 Plants

**Figure 8:**
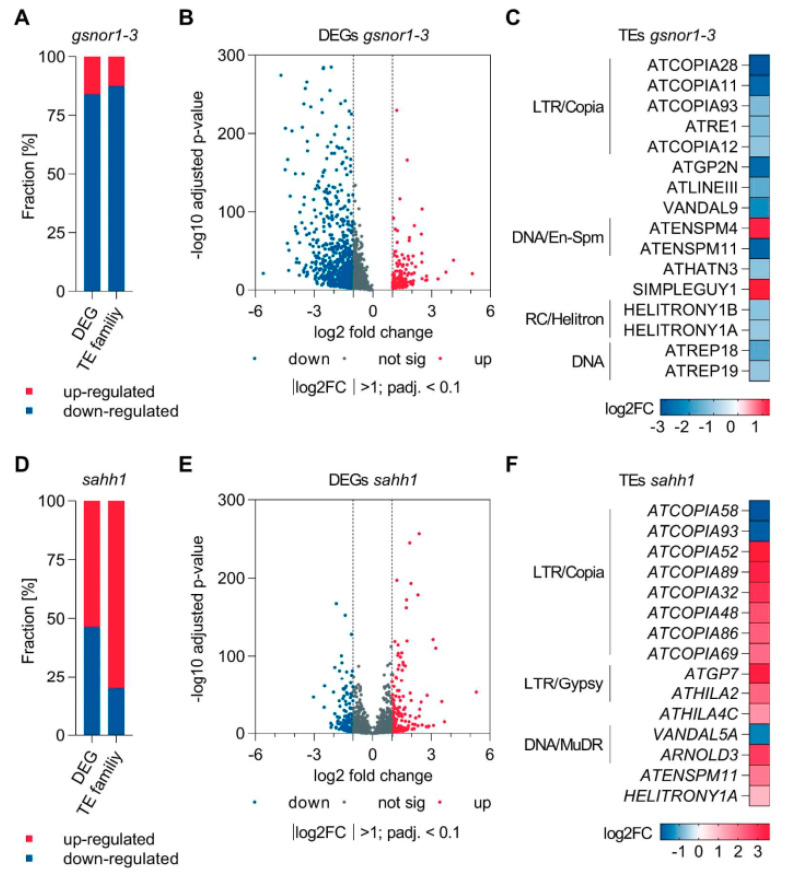
**Mutations in *GSNOR1* and *sahh1* result in transcriptional reprogramming**. (**A**,**D**) Stacked bar plots showing the fraction of significant differentially expressed genes (DEGs) and transposable element (TE) families in *gsnor1-3* and *sahh1*. (**B**,**E**) Volcano plot highlighting significant DEGs in *gsnor1-3* and *sahh1*. Blue and red dots mark significantly decreased or increased expressed genes, respectively. (**C**,**F**) Heat map showing differential expression of TE families in *gsnor1-3* and *sahh1*. If more than one family within a superfamily is differentially expressed, superfamilies are indicated. TEs are classified according to www.arabidopsis.org (accessed on 24 December 2018).

We performed RNA sequencing to link the observed differential DNA methylation in *sahh1* and *gsnor1-3* with gene expression and physiological functions. Among the 1129 differentially expressed genes (DEGs; │log2FC│ > 1, adjusted *p*-value less than 0.1) identified in *gsnor1-3* relative to wt, three quarters (949) were downregulated, and only 180 were upregulated (Figure 8A,B; Supplemental Table S7); similarly, most TE families were downregulated (Figure 8C; Supplemental Table S8). Transcriptional profiling of *sahh1* revealed 394 DEGs which were evenly up- and downregulated (211 vs. 183) (Figure 8D,E; Supplemental Table S9). In contrast, expression of TEs from most TE families was upregulated in *sahh1* (Figure 8D,F; Supplemental Table S10).

RNA was extracted from 4-week-old rosette leaves grown under long-day conditions and harvested 5 h after the day-time start (*n* = 4). Significant criteria: │log2FC│ > 1, adjusted *p*-value less than 0.1. Gene Ontology (GO) term enrichment analysis revealed that among the most significantly enriched molecular functional categories of the upregulated genes in *gsnor1-3* were the “catalytic activity”, “glutathione transferase”, “glycosyltransferase”, and “oxidoreductase activity” categories (Supplemental Table S11). Moreover, biological process categories such as “response to light”, “response to UV”, “cellular response to reactive oxygen species”, “cellular response to oxidative stress”, and “cellular response to nitric oxide” were significantly enriched. According to that, the transcript profile analysis of *gsnor1-3* plants suggests a pre-induced antioxidant system under normal growth conditions, as previously reported for *gsnor* (Ws background [39]). Further, ^•^NO response was enriched among upregulated genes. This is in agreement with the enhanced RSNO/^•^NO level in *gsnor1-3* [34,35]. The majority of downregulated genes in the GO term “biological process” are related to the defense response and response to stress.

Concerning *sahh1*, the terms “DNA-binding transcription factor activity” and “metal ion binding” were the dominant categories among the molecular functions enriched for downregulated genes (Supplemental Table S12). Among biological processes, terms related to “hormones” and “response to chemical” were over-represented. For instance, *LIPOXYGENASE 4* involved in the biosynthesis of the plant hormone jasmonic acid was downregulated, as previously reported [56]. Further, the term anthocyanin-containing compound biosynthesis was found when analyzing upregulated DEGs in *sahh1*, which is in line with previous studies [56].

### 3.6. Integrative Analysis of WGBS and RNA-Seq Data

To test whether differential methylation was associated with differential expression, we looked for associations between the WGBS and RNA-seq datasets (DEG–DMG candidates). The integrative analysis of DMGs and DEGs at the gene level revealed that about 4% of DMGs were differentially expressed (percentages are relative to DMGs). Hypo- and hypermethylation were positively and negatively correlated with transcription (Table 3). Loss of GSNOR function resulted in hypermethylation of up- and downregulated TEs in the CHG context (Supplemental Figure S5). Integrative analysis of differentially methylated TEs and differentially expressed TE families revealed that DNA methylation is negatively associated with TE expression. In detail, TE families ATLINEIII, ATHATN3, and HELITRONY1A were downregulated (expression analysis performed at family level; Supplemental Table S8), and members of those TE families were hypermethylated (Supplemental Table S4).

Metaplot analysis revealed that the DNA methylation levels of the identified up- and downregulated genes in *sahh1* tended to decrease compared to wt (Supplemental Figure S6). An integrative analysis of DMGs and DEGs at the gene level revealed that about 1.7% of DMGs were differentially expressed (percentages are relative to DMGs). Upregulated genes mainly correlated with decreased methylation in their 3kb upstream and genic region (Supplemental Figure S6). Interestingly, downregulated genes were also associated with reduced DNA methylation levels in their 3kb up- or downstream region in *sahh1* (Table 4; Supplemental Figure S6). Hypomethylation in each sequence context was observed in up- and downregulated TEs in *sahh1* (Supplemental Figure S6). 

Integrative analysis of differentially methylated TEs and differentially expressed TE families revealed that DNA hypomethylation caused TE activation. In detail, TE families ATCOPIA89, ATHILA2, and HELITRONY1A were upregulated (expression analysis performed at family level; Supplemental Table S10), and members of those TE families possess hypo-DMRs (Supplemental Table S6). Taken together, there is a low correlation between altered DNA methylation and the expression of protein-coding genes, whereas DNA methylation is principally negatively correlated with TE expression. 

## 4. Discussion

### 4.1. Loss of GSNOR1 Function Results in an Increased Methylation Index

The main function of the methylation cycle is to produce SAM for transmethylation reactions and to recycle the by-product inhibitor SAH [48,49]. The SAM/SAH ratio (MI) is considered as a metabolic indicator of the organismal methylation status [51], since SAM is used as a methyl donor by methyltransferases, and SAH competitively inhibits most of the known SAM-dependent methyltransferases [87]. Loss of GSNOR1 triggered a metabolic reprogramming affecting the methylation cycle by increasing the level of SAM. Since the level of SAH is not altered in *gsnor1-3*, the SAM/SAH ratio consequently increased (Figure 1A,C). In *sahh1*, the level of SAM is also enhanced, but since SAH accumulates stronger, the SAM/SAH ratio is finally decreased in *sahh1*. Surprisingly, metabolites of pathways connected to the methylation cycle (MTA, Cys, GSH) are increased in both plant mutants, concluding that the GSNOR and SAHH1 function is involved in regulating the levels of these metabolites, which also influence methylation processes. In terms of epigenetics, GSH was demonstrated to impact epigenetic mechanisms in the animal system [88]. For instance, the activity of the liver isoform SAMS1 depends on the GSH/GSSG ratio [88], indicating a crosstalk between GSH/GSSG levels and SAM synthesis. Moreover, SAM inhibits demethylase activity in vitro and in cells [89]. However, since SAM is highly unstable, it is not clear whether its in vivo activities are caused by SAM or by SAM metabolites, such as MTA [90]. MTA was shown to affect histone methylation as a histone methyltransferase inhibitor [91]. Furthermore, the combination of metabolic changes might have synergistic effects on the epigenetic landscape.

Interestingly, transcriptomic changes of genes involved in the methylation cycle were not observed in *gsnor1-3* (Supplemental Table S7). We confirmed in vitro S-nitrosation of SAHH1 by GSNO using purified recombinant SAHH1 and plant protein extracts (Supplemental Figure S2A,C). Furthermore, other groups demonstrated that S-nitrosation strongly inhibits SAHH1 activity in vitro [82]. This, at least, raises the possibility that the formation of SAHH1-SNO plays a role in fine-tuning the SAHH1 enzyme activity in respect to epigenetic methylation marks under yet unknown conditions. However, the S-nitrosation of SAHH1 and its influence on the enzyme activity in vivo would certainly require further experimental analysis. 

Interestingly, metabolites of pathways connected to the methylation cycle, such as MTA, Cys, and GSH, were increased in both *gsnor1-3* and *sahh1* (Figure 1D–F), demonstrating that GSNOR and SAHH1 are also important for regulating the levels of these metabolites. 

### 4.2. GSNOR1 Function Is Crucial for the Maintenance of Histone Methylation and DNA Methylation

Several lines of evidence have demonstrated that an altered MI affects histone and DNA methylation in plants and animals ([42,50,51] and references therein). To date, the interconnection between an increased MI and hypermethylation has been rarely reported [92,93], whereas a decreased MI concomitant with a hypomethylated phenotype, as observed in the *sahh1* plants, has been described frequently ([50,51] and references therein). Indeed, a decreased MI predominantly results in loss of H3K9me2 and loss of non-CG methylation, whereas other histone methylation marks, such as H3K27me1 and H3K9me1, and CG methylation are less affected in *Arabidopsis* ([50,51] and references therein). 

Loss of the GSNOR1 function results in global hypermethylation of H3K9me2 and H3.1.K27me2 (Table 1). However, we can only speculate about the exact GSNO/^•^NO-dependent molecular mechanisms regulating the methylation of these histone marks. Besides modulation of the methylation cycle via affecting SAM levels (Figure 1A), GSNO/^•^NO could also regulate the expression of genes encoding for histone modifiers and/or could directly modulate the activity of proteins involved in histone (de-)methylation via GSNO/^•^NO-mediated PTMs. Indeed, several genes involved in histone methylation are differentially expressed in *Arabidopsis* plants with impaired ^•^NO homeostasis or after ^•^NO donor treatment (summarized in Lindermayr et al. [94]). Moreover, regulation of the *Arabidopsis* histone arginine demethylase PRMT5 by S-nitrosation has been reported [95].

Histone methylation is functionally linked to DNA methylation. For instance, H3K9me2 and non-CG methylation are connected by a reinforcing loop, which perpetuates both epigenetic marks catalyzed by CMT2/3 and SUVH4/5/6, respectively [84]. However, SUVH5 and SUVH6 can also bind to DNA that is methylated in the CG context in vitro [96], supporting the view that CG methylation also contributes to H3K9me2 deposition. In addition to CG methylation, it has been known for many years that CHH methylation generated by the RNA-directed DNA methylation pathway is also involved in H3K9me2 deposition [97,98,99,100]. Profiling of cytosine methylation patterns with high resolution by WGBS demonstrated that loss of the GSNOR1 function affects DNA methylation. Although the MI was increased, almost unchanged global mean DNA methylation rates were observed in *gsnor1-3* (Table 2). However, in relation to the genome-wide position of methylated cytosines, rather hyper-DMRs, as opposed to hypo-DMRs, were identified (Figure 5B). In fact, the number of hyper-DMRs was more than 3.8 times that of hypo-DMRs (61 hypo-DMRs; 231 hyper-DMRs). This finding indicates that the GSNOR1 function seems to be important for the hypermethylation of these regions. ^•^NO-induced changes in the expression of genes related to DNA methylation further demonstrated the importance of ^•^NO for DNA (de-)methylation processes (summarized in Lindermayr et al. [94]; Supplemental Table S7). Based on studies in the human/animal field, different effects could contribute to the altered DNA methylation pattern in *gsnor1-3*. For instance, reduced active DNA demethylation could tile the equilibrium of methylation processes toward methylation in *gsnor1-3*. In this context, elevated levels of SAM, as observed in *gsnor1-3* (Figure 1A), counteract active DNA demethylation in human cells [89,101]. Further, mammalian TET enzymes involved in DNA demethylation are inhibited by ^•^NO due to the formation of a nitrosyl–iron complex with their catalytic iron [102]. Similarly, the iron–sulfur-containing ROS1/DME DNA demethylases [103] could be affected by ^•^NO in *gsnor1-3*. The attack of iron–sulfur clusters by ^•^NO [104] has been previously shown. For instance, ^•^NO inhibits aconitase by forming a metal–nitrosyl complex with its iron–sulfur cluster [105]. Further, iron sequestration via DNIC formation may yield reduced iron bioavailability for iron–sulfur cluster assembly. In this context, *Arabidopsis* mutants impaired in the iron–sulfur cluster assembly pathway reveal DNA hypermethylation [106]. Moreover, hypermethylation could be a result of enhanced DMT activity. In this context, increased DMT activity was observed in nuclear protein extracts treated with ^•^NO [107]. 

### 4.3. Alteration in DNA Methylation Does Not Correlate with Gene Expression

Several recent studies indicated a weak association between differential DNA methylation and gene expression changes [108]. For instance, in *Arabidopsis* mutants impaired in the methylation cycle, *mat4* [61] and *ms1* [50], differential DNA methylation of genes was not associated with their expression. Consistent with these findings, differentially expressed genes displayed no significant differences in DNA methylation profiles between *gsnor1-3* and wt. Hence, these results indicate that transcriptional changes occur largely independently of detectable variation in the DNA methylation pattern. In this regard, only 4% of DMGs (genes overlapping with identified DMRs in their genic, 3kb up- and/or downstream region) were differentially expressed. This finding is comparable to previous studies. For instance, about 5% of DMGs were differentially expressed in *Arabidopsis* roots challenged with beet cyst nematode *Heterodera schachtii* [108]. Promotor methylation (3kb upstream region) was typically associated with gene repression; however, in some cases, it enhanced gene transcription in *gsnor1-3* (Table 3). Gene body methylation (between start and stop codons) seems to have a weak effect on gene expression in *Arabidopsis* [109,110], and its function remains enigmatic [111]. 

Nevertheless, constitutive mis-regulation of genes which are not directly targeted by DNA methylation may result from methylation-dependent alteration in the transcriptional networks [112]. The linkage between DEGs not targeted by differential DNA methylation and methylation-dependent alteration in the transcriptional network [62,112] is exemplified at the *PR1* gene. The *PR1* transcript is upregulated in mutants globally defective in the maintenance of CG (*met1*) or non-CG methylation (*ddc*) [112], whereas *PR1* is downregulated in hypermethylated *35S::MS1* plants [62]. Likewise, *PR1* expression is reduced (Supplemental Table S7) and delayed [34] in *gsnor1-3*. Notably, mutants globally defective in DNA methylation were markedly resistant to *Pst* [112], whereas plants with an increased DNA methylation level (*35S::METS1*; *Arabidopsis* plants overexpressing *MS1*) and *gsnor1-3* showed attenuated resistance to *Pst* [34,62]. 

Besides altered DNA methylation levels, transcriptional changes are probably also caused by the pleiotropic effects of an impaired GSNOR1 function. For instance, loss of the GSNOR1 function caused the differential expression of several transcription factors (Supplemental Table S7). Further, proteins involved in transcriptional regulation were identified as targets for S-nitrosation [33]. Moreover, loss of the GSNOR1 function caused enhanced global levels of H3K27me2 (Table 1), which is usually highly enriched at the promoter of inactive genes [113]. Other reasons why loss of the GSNOR1 function induces transcriptional changes could be the modulation of the chromatin structure by other epigenetic mechanisms. For instance, non-coding miscellaneous RNAs are differentially expressed in response to GSNO [114]. In general, non-coding RNAs are regulators of gene expression by a variety of mechanisms such as chromatin remodeling, or they regulate gene expression at the transcriptional or post-transcriptional levels. Furthermore, transcriptional changes could be linked to the proximity of differentially methylated TEs to DEGs [108]. 

### 4.4. GSNOR1 Regulates Demethylation and Expression of TEs and Stress-Responsive Genes

GSNOR1 activity is required for the reduction in H3K9me2. H3K9me2 plays important roles in plant environmental stress response [115]. For instance, gene expression induced by ABA and salt stress is associated with the reduction in gene repression marks, such as H3K9me2, at ABA and abiotic stress-responsive genes [116]. In this context, lowering the H3K9me2 level at stress-related genes might be a regulatory mechanism of GSNOR1 to activate the stress response. Moreover, the repressive histone mark H3K9me2 is associated with TE silencing. Repression of TEs is required to guarantee genome stability. Therefore, TEs are generally located in transcriptionally silenced heterochromatic regions marked by DNA methylation and repressive histone modifications, such as H3K9me2 [43,115]. In *gsnor1-3*, DNA methylation differs in the TE-rich pericentromeric region from wt (Figure 4). Indeed, parts are hyper- and hypomethylated. However, the genomic annotation of the identified DMRs resulted in mainly hypermethylated TEs (Figure 6A,D). Among them, LTR/Copia- and Line/L1-type TEs, predominantly regulated through H3K9me2 and non-CG DNA methylation pathways [117], but also LTR/Gypsy-type TEs, predominantly regulated by H3K27me1 methylation [117], were found. Consistent with the enhanced DNA methylation, the RNA-seq data indicate that TEs (expression analysis performed at family level) were mainly repressed in the *gsnor1-3* mutant (Figure 8A–C). The expression of transposons under plant stress, such as heat, cold, drought, wounding, viruses, and pathogens [118], is a well-known phenomenon [119,120,121,122,123,124,125,126]. According to McClintock [127], boosting the expression and transposition activity of TEs in environmental stress conditions results in extensive genomic re-structuring, which finally facilitates the adaptation of species and populations to a changing environment [128]. Moreover, the TEs closely associated with genic regions could be involved in directly reprogramming transcriptional networks, affecting the expression profiles of individual genes and fine-tuning the host response to specific stimuli [129,130]. In this context, the impaired plant disease responses [34,131] and the heat sensitivity [35] of GSNOR1-deficient *Arabidopsis* could, at least, be partly based on the reduced activation of TEs. 

Interestingly, the GSNOR1 function is also required for the demethylation and expression of several stress-responsive genes, e.g., Flotillin-like protein1 and 2 (AT5G25250, AT5G25260), which are involved in the UV stress response, or cytochrome P450 94C1 (AT2G27690), which is involved in the wounding response (Table 3). Plant flotillins are a subgroup of the SPFH domain protein superfamily, consisting of three proteins, FLOT1, FLOT2, and FLOT3, in *A. thaliana*. *FLOT* genes respond differentially to different types of abiotic and biotic stresses, nutrient depletion, and phytohormones [132,133].

Cytochrome P450 94C1 encodes an enzyme involved in jasmonoyl-L-isoleucine (JA-Ile) oxidation. Jasmonic acid (JA) is an important signaling hormone exhibiting a broad spectrum of physiological activities in growth and development. JA also fulfills an important signaling function in plant defense, particularly the defense against insect herbivores and necrotrophic pathogens. In particular, the conjugate of jasmonate and isoleucine (JA-Ile) is a major regulator which controls gene expression and production of secondary metabolites after (a)biotic challenges. The two cytochromes P450 94B3 and 94C1 catalyze two successive oxidation steps of JA-Ile for catabolic turnover [134,135]. The oxidized derivatives of JA-Ile accumulate in wounded *Arabidopsis* leaves. CYP94C1 catalyzes the oxidation of 12OH-JA-Ile to 12C00H-JA-Ile, and its transcripts accumulate in response to stress and wounding [136]. However, plants overexpressing CYP94C1 display a strongly impaired defense gene induction as well as reduced disease resistance [135], suggesting that a coordinated turnover of JA-Ile is essential for an effective stress response. In this context, the reduced expression of CYP94C1 in *gsnor1-3* might be responsible for herbivory susceptibility, as demonstrated in GSNOR-silenced *Nicotiana attenuata* [137]. 

**Figure 9:**
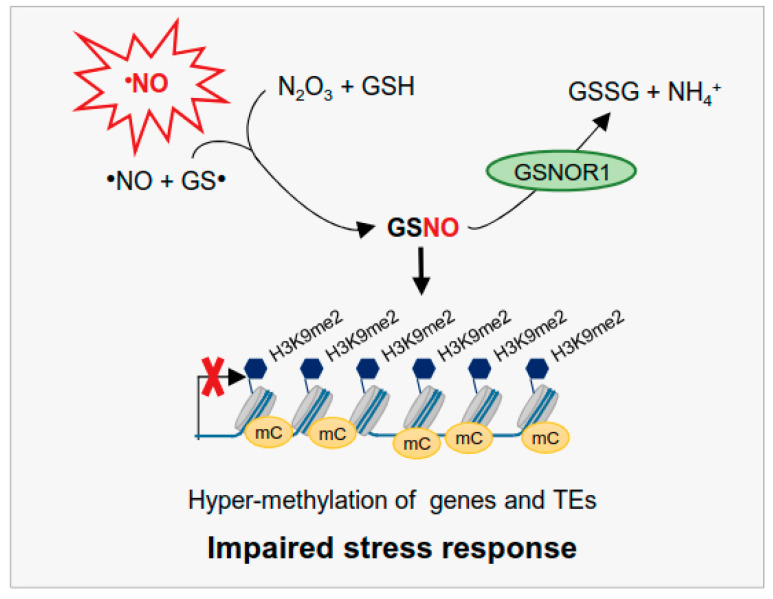
Proposed model illustrating the function of GSNOR1 in regulating methylation processes and expression of TEs and stress-responsive genes. ^•^NO is endogenously produced under physiological conditions [18], and GSNO, as a more stable redox form of ^•^NO, is formed and promotes methylation of H3K9 and DNA. Hypermethylation of TEs and stress-responsive genes results in impaired stress response. Since GSNO is enzymatically degraded by GSNOR1, GSNOR1 activity positively affects stress response by promoting expression of TEs and stress-responsive genes.

In conclusion, the GSNOR1 function is required for a controlled processing of the methylation cycle, for a reduction in the repressive H3K9me2 histone mark, and for TE activation to enable an effective stress response (Figure 9). These findings present a new function of ^•^NO as an epigenetic regulator and provide a new insight into ^•^NO signaling in plants. 

## 5. Conclusions

In this study, we demonstrated that the GSNOR1 function is required for SAM homeostasis, and, consequently, loss of GSNOR1 activity affects transmethylation reactions. We observed a significant global increase in the repressive H3K9me2 mark in *gsnor1-3*. H3K9me2-modified chromatin regions tightly correlate with methylated DNA regions. Whole-genome bisulfite sequencing and transcriptome analyses revealed enhanced DNA methylation and reduced expression of TEs and stress-responsive genes in *gsnor1-3*. This impaired expression of TEs and stress-responsive genes is in accordance with described susceptibility of *gsnor1-3* to e. g. pathogen infection and heat stress. In conclusion, our data suggest that GSNOR1 function is required to reduce the level of the repressive chromatin mark H3K9me2 and DNA methylation at distinct TEs and stress-responsive genes to enable effective stress response. 

## Figures and Tables

**Table 1:** Histone H3K9me2 methylation level is altered in *gsnor1-3* and *sahh1*.

Motif	Sequence of Peptide	Mean % Abundance ± SD		
		*sahh1*	Col-0/wt	*gsnor1-3*
H3.K4_noPTM	TKQTAR	42.81 ± 1.81	43.64 ± 3.12	42.70 ± 3.94
H3.K4me1	TKme1QTAR	56.71 ± 1.75	55.71 ± 2.56	56.43 ± 3.63
H3.K4me2	TKme2QTAR	0.21 ± 0.05	0.29 ± 0.24	0.40 ± 0.16
H3.K4me3	TKme3QTAR	0.27 ± 0.08	0.36 ± 0.3	0.47 ± 0.19
H3.K9_K14_noPTM	KSTGGKAPR	42.62 ± 0.65 **	39.68 ± 0.26	37.09 ± 0.78 **
H3.K9ac	KacSTGGKAPR	1.97 ± 0.31	1.55 ± 0.18	1.58 ± 0.10
H3.K14ac	KSTGGKacAPR	27.07 ± 0.79	27.02 ± 0.19	27.46 ± 0.56
H3.K9ac_K14ac	KacSTGGKacAPR	3.02 ± 0.12	3.13 ± 0.13	2.85 ± 0.20
H3.K9me1_K14ac	Kme1STGGKacAPR	1.17 ± 0.06	1.13 ± 0.08	1.24 ± 0.14
H3.K9me2_K14ac	Kme2STGGKacAPR	0.16 ± 0.03	0.16 ± 0.06	0.22 ± 0.06
H3.K9me3_K14ac	Kme3STGGKacAPR	0.02 ± 0.00	0.03 ± 0.02	0.03 ± 0.01
H3.K9me1	Kme1STGGKAPR	20.01 ± 0.34	21.31 ± 0.61	22.14 ± 0.50
H3.K9me2	Kme2STGGKAPR	3.88 ± 0.09 ***	5.83 ± 0.38	7.17 ± 0.41 **
H3.K9me3	Kme3STGGKAPR	0.10 ± 0.02	0.17 ± 0.03	0.22 ± 0.02
H3.K18_K23_noPTM	KQLATKAAR	60.62 ± 0.93 *	62.46 ± 0.11	62.72 ± 0.83
H3.K18ac	KacQLATKAAR	24.33 ± 0.30 **	22.82 ± 0.25	22.17 ± 0.57
H3.K23ac	KQLATKacAAR	6.90 ± 0.35	6.82 ± 0.08	7.14 ± 0.22
H3.K18ac_K23ac	KacQLATKacAAR	8.15 ± 0.40	7.90 ± 0.09	7.97 ± 0.25
H3.1.K27_K36_K37_noPTM	KSAPATGGVKKPHR	10.19 ± 1.33	9.55 ± 1.09	9.22 ± 0.83
H3.1.K27ac	KacSAPATGGVKKPHR	0.18 ± 0.01	0.17 ± 0.02	0.17 ± 0.01
H3.1.K36ac	KSAPATGGVKacKPHR	0.15 ± 0.01	0.14 ± 0.01	0.13 ± 0.01
H3.1.K27ac_K36me2	KacSAPATGGVKme2KPHR	0.08 ± 0.01	0.07 ± 0.01	0.09 ± 0.03
H3.1.K27ac_K36me3	KacSAPATGGVKme3KPHR	0.78 ± 0.14	0.76 ± 0.09	0.63 ± 0.03
H3.1.K27me2_K36ac	Kme2SAPATGGVKacKPHR	0.16 ± 0.02	0.16 ± 0.02	0.14 ± 0.02
H3.1.K27me3_K36ac	Kme3SAPATGGVKacKPHR	0.08 ± 0.04	0.07 ± 0.01	0.08 ± 0.04
H3.1.K27me1	Kme1SAPATGGVKKPHR	49.45 ± 3.87	49.40 ± 3.38	43.24 ± 0.26
H3.1.K27me2	Kme2SAPATGGVKKPHR	18.68 ± 1.87	19.48 ± 1.46	24.08 ± 0.77 *
H3.1.K27me3	Kme3SAPATGGVKKPHR	6.53 ± 1.63	6.68 ± 1.28	7.74 ± 0.24
H3.1.K36me1	KSAPATGGVKme1KPHR	2.27 ± 0.36	2.32 ± 0.29	2.85 ± 0.78
H3.1.K36me2	KSAPATGGVKme2KPHR	1.49 ± 0.05	1.41 ± 0.08	1.63 ± 0.26
H3.1.K36me3	KSAPATGGVKme3KPHR	9.97 ± 1.76	9.78 ± 1.43	10.22 ± 0.59

Abundance of histone methylation and acetylation marks on histone H3 in 4-week-old *Arabidopsis* plants as determined by LC-MS. Relative abundance of 31 PTMs involving lysine acetylation and methylation marks on histone H3 in 4-week-old rosette leaves grown under long-day conditions and harvested 5 h after the day-time start from wt, *sahh1*, and *gsnor1-3* plants. Statistics: values are the relative abundance of each histone motif at each peptide and represent the mean ± SD (*n* = 3). * (*p* < 0.05), ** (*p* < 0.01), and *** (*p* < 0.001) represent significant differences between wt and mutant lines (ANOVA, Dunnett´s multiple comparisons test). Statistical analysis was performed with GraphPad Prism version 7.05. For calculation of motif abundance, refer to Feller et al. [76]. The motif identifier name contains the PTM type and position. H3.K4me1: abundance of mono-methylation on K4 relative to H3.K4me2, H3.K4me3, and H3.K4noPTM. Kac, lysine acetylation; Kme1, lysine mono-methylation; Kme2, lysine di-methylation; Kme3, lysine tri-methylation; noPTM, peptide without PTM.

**Table 2:** Mean methylation rates per context (±SD) as analyzed by WGBS (*n* = 2). Mean methylation rates in CG, CHG, or CHH context (H = A, C, or T) in Col-0/wt, *sahh1*, and *gsnor1-3* were calculated from cytosines that were covered by at least 5 reads.

	CG	CHG	CHH
Col-0/wt	22.90 ± 2.45%	6.38 ± 1.28%	1.54 ± 0.27%
*gsnor1-3*	20.32 ± 0.19%	5.59 ± 0.07%	1.38 ± 0.03%
*sahh1*	19.43 ± 1.18%	3.11 ± 0.26%	0.95 ± 0.10%

**Table 3:** Integrative analysis of DMGs and DEGs in *gsnor1-3*.

**Overlap of Significantly Downregulated Genes with DMGs**								
**Chr**	**Start**	**bp**	**CH_3_**	**Feature**	**Gene ID**	**log2FC**	**padj.**	**Description**
Chr5	8751681	42	+	3kb down	AT5G25250	−3.37	2.0 × 10^−266^	Flotillin-like protein 1 (UV-stress)
Chr5	9309455	206	−	3kb up	AT5G26690	−3.35	2.3 × 10^−23^	Heavy metal-associated isoprenylated plant protein 2 (stress response; but not much data available)
Chr2	11812888	185	+	3kb down	AT2G27690	−2.94	7.6 × 10^−36^	Cytochrome P450 94C1 (jasmonoyl-L-isoleucine; wounding)
Chr5	8751681	42	+	3kb up	AT5G25260	−2.80	2.0 × 10^−64^	Flotillin-like protein 2 (UV-stress)
Chr2	9741371	43	+	in gene	AT2G22880	−2.24	3.8 × 10^−12^	At2g22880 (Hypoxia, UV-stress, salt, wounding)
Chr2	15110344	63	+	3kb up	AT2G35980	−2.07	1.7 × 10^−8^	NDR1/HIN1-like protein 10 (Hypoxia, salt, biotic stress)
Chr2	18325130	77	+	3kb up	AT2G44380	−2.05	1.8 × 10^−8^	At2g44380 (biotic stress)
Chr1	24395763	100	+	3kb up	AT1G65610	−1.82	4.4 × 10^−6^	Endoglucanase 7 (biotic stress)
Chr5	5767502	32	-	3kb up	AT5G17490	−1.56	6.8 × 10^−10^	DELLA protein RGL3 (wounding, cold)
Chr3	22556563	37	+	3kb down	AT3G60966	−1.45	1.8 × 10^−4^	RING/U-box superfamily protein
Chr2	18325130	77	+	3kb up	AT2G44400	−1.39	1.1 × 10^−3^	Cysteine/Histidine-rich C1 domain family protein
Chr1	27068879	85	+	3kb down	AT1G71910	−1.34	7.2 × 10^−6^	At1g71910
Chr2	3304271	210	−	in gene	AT2G07774	−1.31	4.1 × 10^−5^	unknown protein
Chr5	18136940	44	+	3kb up	AT5G44920	−1.21	2.5 × 10^−3^	TIR domain-containing protein
Chr5	18136984	64	+	3kb up	AT5G44920	−1.21	2.5 × 10^−3^	TIR domain-containing protein
Chr3	3063382	181	−	in gene	AT3G09960	−1.21	4.7 × 10^−3^	Calcineurin-like metallo-phosphoesterase superfamily protein
Chr5	18779966	240	+	in gene	AT5G46295	−1.19	2.3 × 10^−4^	Transmembrane protein
Chr5	18780206	180	+	3kb up	AT5G46295	−1.19	2.3 × 10^−4^	Transmembrane protein
Chr2	12426536	39	−	3kb down	AT2G28940	−1.17	1.7 × 10^−17^	At2g28940
Chr1	4123656	44	−	3kb up	AT1G12160	−1.13	2.0 × 10^−4^	Flavin-containing monooxygenase FMO GS-OX-like 1
Chr1	21823145	288	−	3kb down	AT1G59124	−1.08	2.9 × 10^−19^	Probable disease resistance protein RF45
**Overlap of Significantly Upregulated Genes with DMGs**								
**Chr**	**Start**	**bp**	**CH_3_**	**Feature**	**Gene Id**	**log2FC**	**padj.**	**Description**
Chr5	7376314	54	−	3kb up	AT5G22300	1.82	1.3 × 10^−39^	Bifunctional nitrilase/nitrile hydratase NIT4 (UV stress, biotic stress)
Chr3	9173846	95	−	3kb up	AT3G25190	1.30	2.7 × 10^−4^	Vacuolar iron transporter homolog 2.1 (biotic stress)
Chr5	17145940	99	+	3kb up	AT5G42760	1.29	1.8 × 10^−8^	Leucine carboxyl methyltransferase
Chr4	13766210	58	+	3kb up	AT4G27570	1.02	1.1 × 10^−2^	UDP-glycosyltransferase 79B3 (jasmonate, cold)
Chr5	9637396	186	+	3kb up	AT5G27330	1.02	2.3 × 10^−11^	Prefoldin chaperone subunit family protein

The methylation status in *gsnor1-3* compared to wt is given as (−) and (+) referring to hypo- and hypermethylation, respectively. DMRs are annotated with genomic features (3kb up- or downstream, and in gene). Abbreviations: Chr, chromosome; start, DMR start position; bp, length of overlapping DMR with genomic feature. Statistics for RNA-seq: │log2FC│ > 1, adjusted *p*-value less than 0.1.

**Table 4:** Integrative analysis of DMGs and DEGs in *sahh1*.

**Overlap of Significantly Downregulated Genes with DMGs**								
**Chr**	**Start**	**bp**	**CH_3_**	**Feature**	**Gene ID**	**log2FC**	**padj.**	**Description**
Chr5	19178939	108	−	3kb up	AT5G47230	−1.94	1.3 × 10^−25^	Ethylene responsive element binding factor 5 ERF5
Chr5	5907343	107	−	3kb up	AT5G17860	−1.88	1.3 × 10^−8^	Cation/calcium exchanger 1
Chr2	18497356	377	−	3kb down	AT2G44840	−1.79	9.7 × 10^−16^	Ethylene-responsive transcription factor 13 ERF13
Chr1	13837861	133	−	3kb up	AT1G36622	−1.51	4.6 × 10^−5^	Transmembrane protein
Chr1	13837994	23	−	3kb up	AT1G36622	−1.51	4.6 × 10^−5^	Transmembrane protein
Chr5	7261113	306	−	3kb up	AT5G21960	−1.26	2.2 × 10^−3^	Ethylene-responsive transcription factor ERF016
Chr5	16023667	82	−	3kb up	AT5G40010	−1.24	8.2 × 10^−4^	AAA-ATPase ASD, mt
Chr1	26140005	248	−	3kb up	AT1G69530	−1.08	1.7 × 10^−128^	Expansin
Chr4	14031509	89	−	3kb down	AT4G28350	−1.07	8.3 × 10^−10^	Probable L-type lectin-domain containing receptor kinase VII.2
**Overlap of Significantly Upregulated Genes with DMGs**								
**Chr**	**start**	**bp**	**CH_3_**	**Feature**	**Gene ID**	**log2FC**	**padj.**	**Description**
Chr5	9206475	54	−	in gene	AT5G26270	5.31	3.0 × 10^−53^	unknown protein
Chr3	20260251	114	−	in gene	AT3G54730	3.69	1.3 × 10^−15^	Putative transmembrane protein At3g54730
Chr3	20260365	94	−	in gene	AT3G54730	3.69	1.3 × 10^−15^	Putative transmembrane protein At3g54730
Chr3	20260459	7	+	3kb down	AT3G54730	3.69	1.3 × 10^−15^	Putative transmembrane protein At3g54730
Chr5	18208166	230	−	in gene	AT5G45095	3.12	7.8 × 10^−11^	Putative uncharacterized protein
Chr1	12851246	141	−	3kb up	AT1G35140	2.38	1.2 × 10^−257^	Protein EXORDIUM-like 1
Chr4	6431517	56	+	in gene	AT4G10380	1.44	6.9 × 10^−10^	At4g10380
Chr2	12887310	93	−	3kb down	AT2G30210	1.44	9.8 × 10^−4^	Laccase-3
Chr2	13160854	47	−	3kb up	AT2G30930	1.43	2.3 × 10^−99^	Expressed protein
Chr1	3980123	55	−	in gene	AT1G11785	1.31	2.6 × 10^−3^	Putative uncharacterized protein
Chr3	9173846	95	−	3kb up	AT3G25190	1.29	6.6 × 10^−4^	Vacuolar iron transporter homolog 2.1
Chr3	21509510	77	−	3kb up	AT3G58070	1.14	2.0 × 10^−3^	GIS
Chr3	20206910	10	−	3kb up	AT3G54580	1.01	4.4 × 10^−2^	Proline-rich extensin-like family protein

The methylation status in ina*sahh1* compared to wt is given as (−) and (+) referring to hypo- and hypermethylation, respectively. DMRs are annotated with genomic features (3kb up- or downstream, and in gene). Abbreviations: Chr, chromosome; start, DMR start position; bp, length of overlapping DMR with genomic feature. Statistics for RNA-seq: │log2FC│ > 1, adjusted *p*-value less than 0.1.

## Data Availability

All the data analyzed for this manuscript are included. The analyzedraw data are available upon reasonable request to the corresponding author.

## Supplementary Materials

The following are available online at https://www.mdpi.com/article/10.3390/antiox10071128/s1. Supplemental Table S1. Oligonucleotides used for the characterization of transgenic lines and cloning. Supplemental Table S2. DMRs identified in *gsnor1-3* and *sahh1* compared to wt. Supplemental Table S3. DMGs identified in *gsnor1-3* compared to wt. Supplemental Table S4. DMRs overlapping with TEs in *gsnor1-3*. Supplemental Table S5. DMGs identified in *sahh1* compared to wt. Supplemental Table S6. DMRs overlapping with TEs in *sahh1*. Supplemental Table S7. DEGs identified in *gsnor1-3* compared to wt. Supplemental Table S8. TE families differentially expressed in *gsnor1-3* compared to wt. Supplemental Table S9. DEGs identified in *sahh1* compared to wt. Supplemental Table S10. TE families differentially expressed in *sahh1* compared to wt. Supplemental Table S11. List of GO terms significantly enriched in the set of DEGs in *gsnor1-3*. Supplemental Table S12. List of GO terms significantly enriched in the set of DEGs in *sahh1*. *Supplemental Figure Legend:* Supplemental Figure S1. Loss of GSNOR1 function results in an increased RSNO content under basal conditions. Supplementary Figure S2. SAHH1 is S-nitrosated and inhibited by GSNO. Supplemental Figure S3. PCR-based genotyping of transgenic lines harboring *TS-GUS* insertion and *sahh1* or *gsnor1-3* mutation. Supplemental Figure S4. Annotation of DMRs to genomic features. Supplemental Figure S5. DNA methylation is poorly correlated with gene expression differences in *gsnor1-3*. Supplemental Figure S6. DNA methylation is poorly correlated with gene expression differences in *sahh1*.
