# Supplementary material for: GSNOR Contributes to Demethylation and Expression of Transposable Elements and Stress-Responsive Genes
Source: Antioxidants (Basel). 2021 Jul 15;10(7):1128. doi: 10.3390/antiox10071128 (PMC8301139; doi:10.3390/antiox10071128)
Supplement: Supplementary file 1 [file antioxidants-10-01128-s001.zip › antioxidants-1273620-supplementary.pdf]

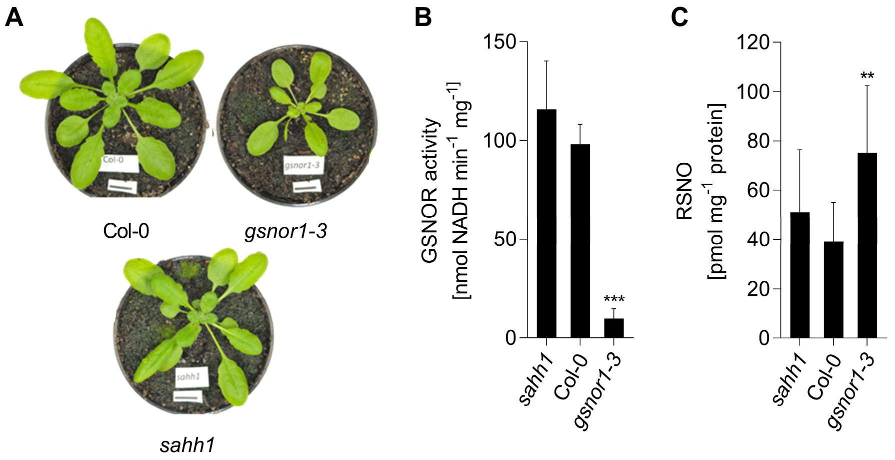

Supplementary Figure S1. Loss of *At*GSNOR1 function results in an increased RSNO content under basal conditions.

(A) Phenotype of 4-week-old *Arabidopsis* mutants. **(B)** GSNOR activity was determined by NADPH consumption in the presence of GSNO (n = 3-5). **(C)** RSNO content was determined by triiodide-dependent ozone-based chemiluminescence (n ≥ 5). Both analyses were measured in 4-week-old rosette leaves grown under long-day condition harvested 5 h after day-time start. Statistics: Values represent the mean ± SD of at least three independent experiments. Grubb´s outlier-test (α = 0.05) was performed. **($p<0.01$) and ***($p<0.001$) represents significant differences between wild-type and mutants (ANOVA with Dunnett´s multiple comparisons test).

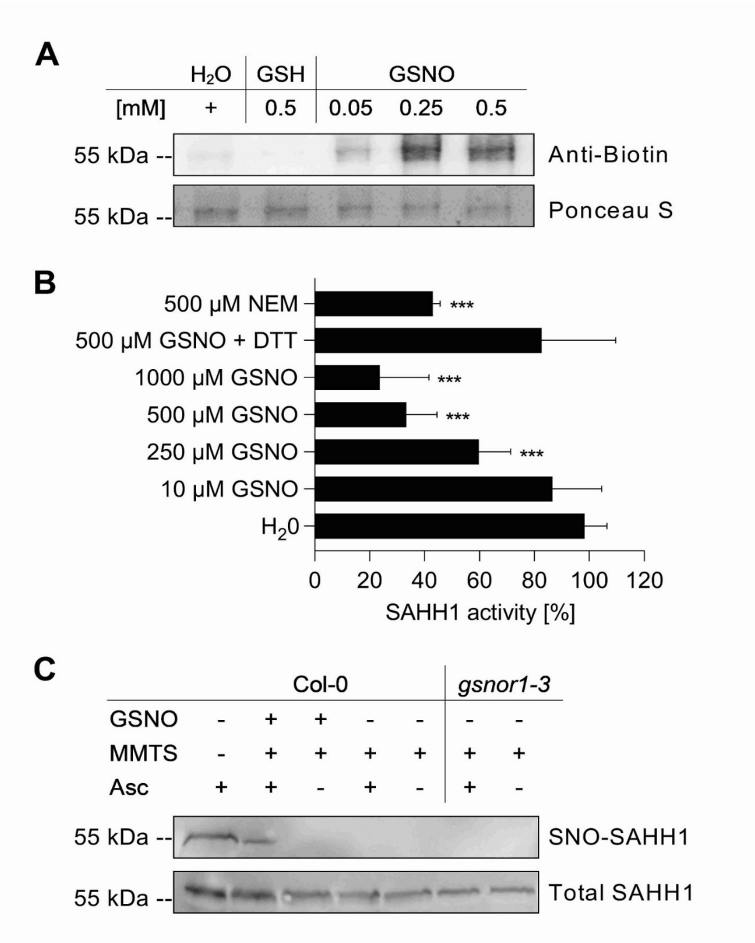

Supplementary Figure S2. SAHH1 is S-nitrosated and inhibited by GSNO. (A) *In vitro* S-nitrosation of SAHH1. 10 µg of purified recombinant His-tagged SAHH1 (56 kDa) was treated with water, GSH, and increasing concentrations of GSNO and subjected to the biotin switch assay using ascorbate (Asc) as reducing agent. Electrophoretically separated proteins were transferred onto a nitrocellulose membrane. Ponceau S staining of blotted proteins demonstrate sample loading. Biotinylated SAHH1 was detected by an alkaline phosphatase-conjugated anti-Biotin IgG followed by BCIP/NBT chromogenic visualization. **(B)** Concentration dependent inhibition of SAHH1 by GSNO *in vitro*. SAHH1 produced in *E. coli* was treated with water (control), GSNO, and NEM with indicated concentration. After desalting, SAHH1 activity was measured. For restoring of SAHH1 activity, 10 mM DTT was added to the GSNO inhibited enzyme and then desalted. Statistics: Values are expressed as percentage of the control activity (at 0 mM: 0.44-0.89 nmol SAH min^{-1} $µg^{-1}$ varied among independent purification) and represent the mean ± SD of at least three independent preparations of recombinant SAHH1 (n = 3-7). Grubb´s outlier-test (α = 0.05) was performed. ***(p<0.001) represents significant differences between non-treated and treated SAHH1 enzyme (ANOVA, Dunnett´s multiple comparisons test). Statistical analysis was performed with GraphPad Prism version 7.05. **(C)** S-Nitrosation of SAHH1 (54 kDa) in wt and *gsnor1-3* leaf extracts. Leaf extracts from Col-0/wt plants (+/-GSNO) and *gsnor1-3* were subjected to the RSNO-RAC. *In vivo* S-nitrosation of SAHH1 was not detected. Total SAHH1 protein ensures equal protein loading. The assay was repeated thrice with similar results.

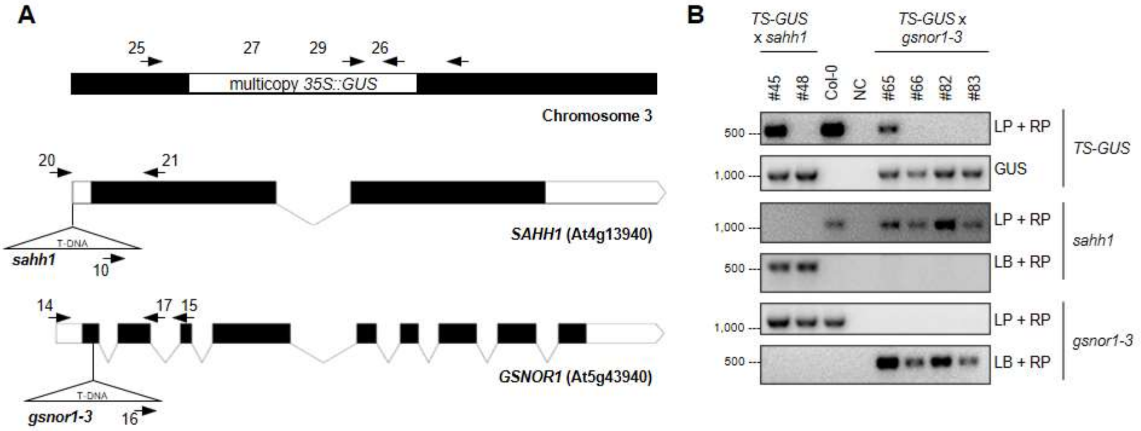

Supplementary Figure S3. PCR-based genotyping of transgenic lines harboring *TS-GUS* insertion and *sahh1* or *gsnor1-3* mutation.

(A) The diagram illustrates the positions of the single insert of a multicopy *35S::GUS* transgene. Black box, Chromosome 3; white box, *GUS* locus. Below the position of the T-DNA insertion sites for *sahh1* and *gsnor1-3* are shown. Black box, coding region; open box, untranslated region; solid black line, intron. The T-DNA insertion of the mutant line *sahh1* are located in the 5´UTR. The position of the T-DNA insertion in *gsnor1-3* is located in the first exon. **(B)** PCR-based genotyping resulted in homogeneity for *TS-GUS* x *sahh1 a*nd *TS-GUS* x *gsnor1-3* for #48, and #66, #82, #83, respectively. Amplicon length, primers, and PCR conditions are listed Supplemental Table S3. DNA ladder (bps) is indicated. Abbreviation: *TS-GUS*, multicopy *35S::GUS* insertion; LP/RP, left/right border genomic primer; LB T-DNA border primer.

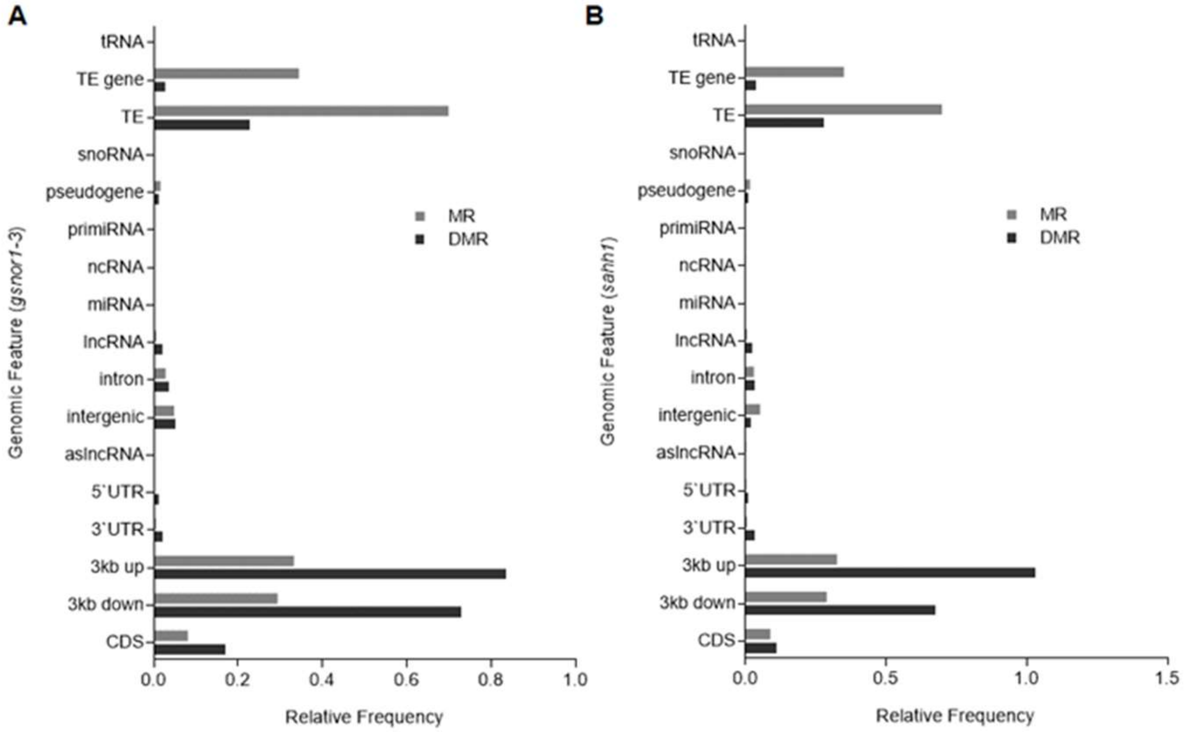

Supplementary Figure S4. Annotation of DMRs to genomic features.

Genomic context of MRs and DMRs identified in *gsnor1-3* **(A)** and *sahh1* **(B)**. TAIR 10 was used for annotation of genomic elements. MRs and DMRs were assigned to the following annotated elements: coding sequence (CDS), 3kb up- and 3kb down-stream of protein coding genes, 5' UTR, 3' UTR, as-lncRNA, intergenic, intron, lncRNA, miRNA, ncRNA, pri-miRNA, pseudogene, snoRNA, transposable element (TE), TE gene, and tRNA.

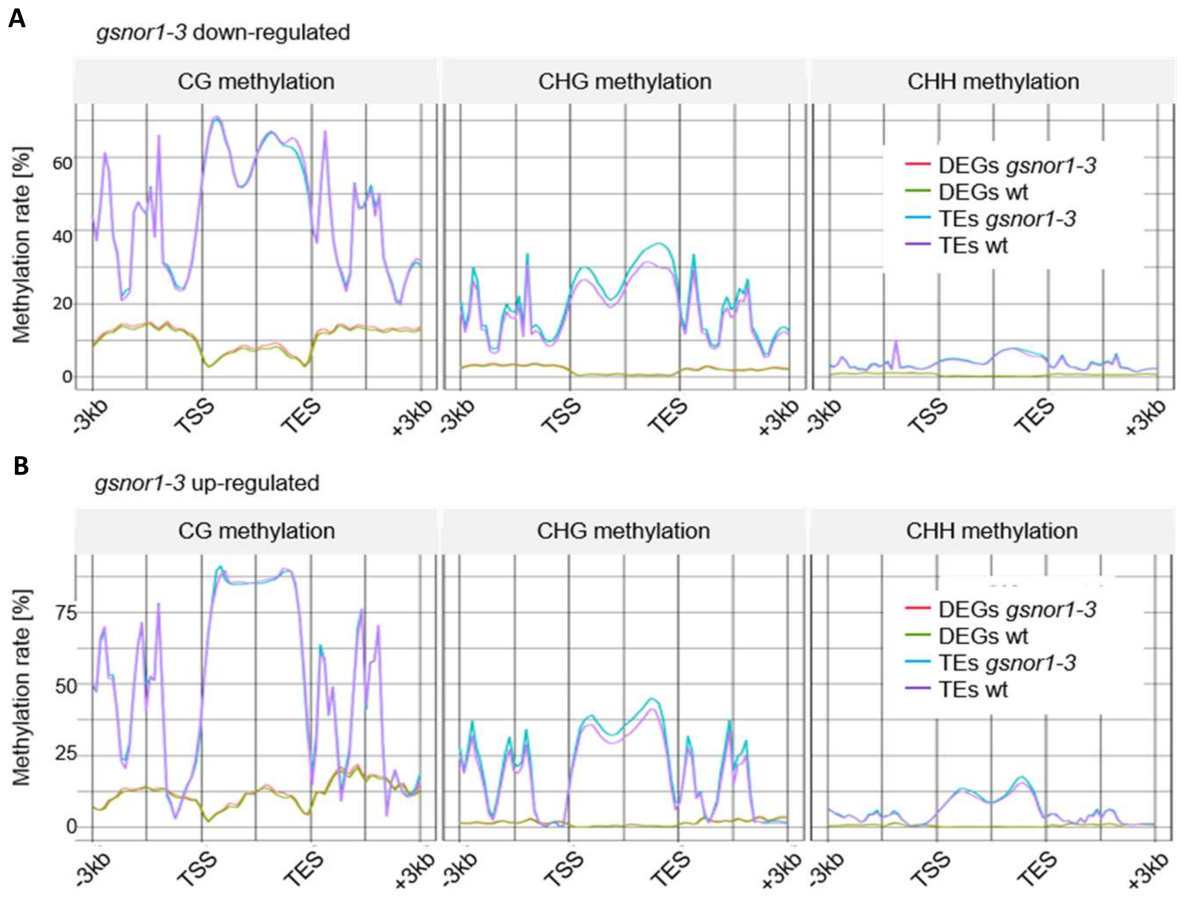

Supplementary Figure S5. DNA methylation is poorly correlated with gene expression differences in *gsnor1-3*.

Average levels of CG, CHG, and CHH methylation over DEGs (identified as **(A)** down- or **(B)** up-regulated in *gsnor1-3*) and differentially expressed TE families as identified by RNA-seq are plotted from 3kb up- to 3kb down-stream. Abbreviations: TSS, transcriptional start site; TES, transcriptional end site; DEGs, differential expressed genes.

A

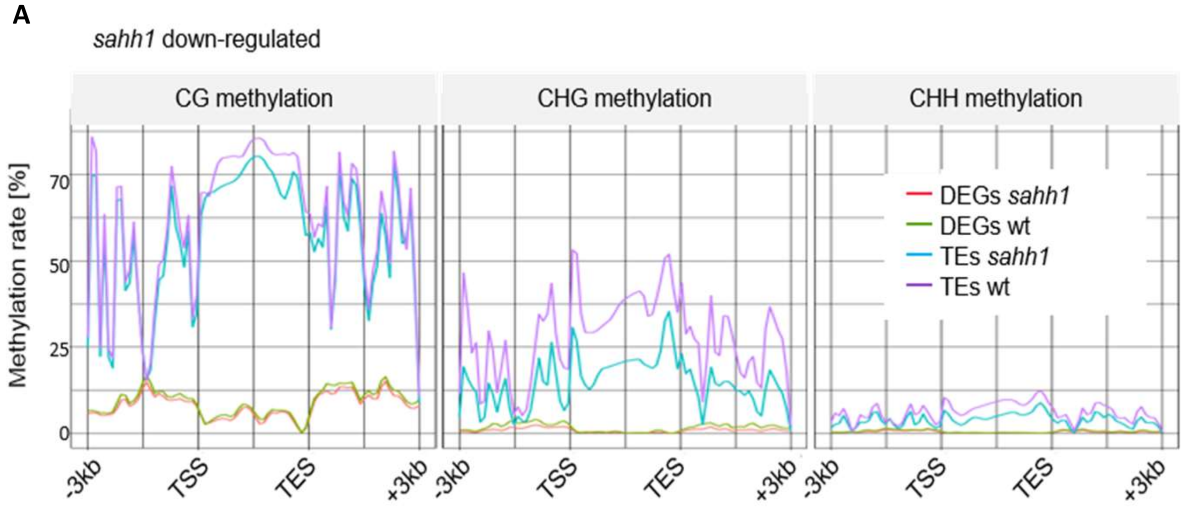

B

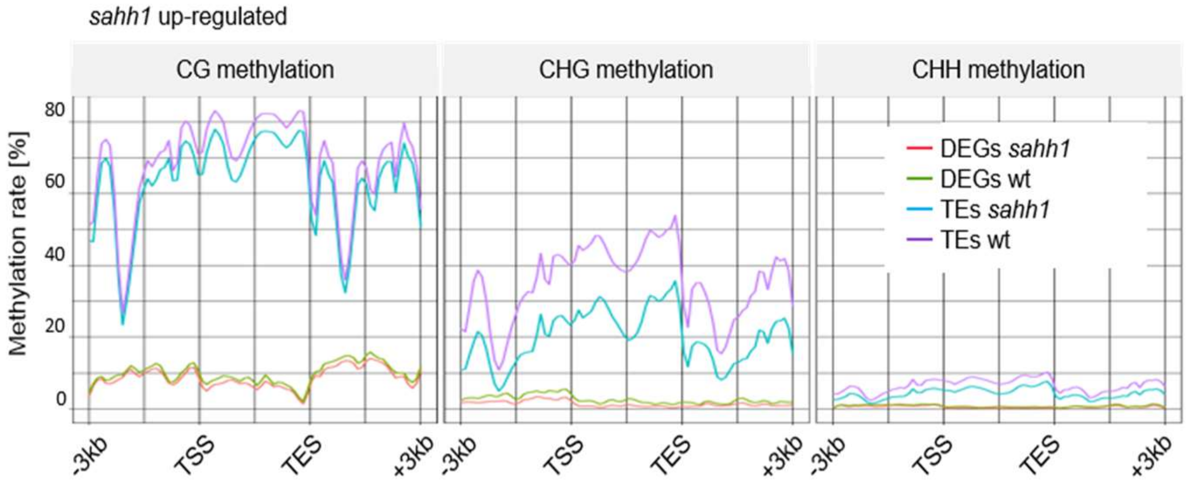

Supplementary Figure S6. DNA methylation is poorly correlated with gene expression differences in *sahh1*.

Average levels of CG, CHG, and CHH methylation over DEGs (identified as **(A)** down- or **(B)** up-regulated in *sahh1*) and differentially expressed TE families as identified by RNA-seq are plotted from 3kb up- to 3kb down-stream. Abbreviations: TSS, transcriptional start site; TES, transcriptional end site; DEGs, differential expressed genes.

Supplemental Methods

Heterologous production of *At*SAHH1

*At*SAHH1 was produced as N-terminal His_6-tagged fusion protein in *E. coli* BL21 (DE3) carrying the pDEST17/*T7::His₆-AtSAHH1* plasmid, which encodes the full-length cDNA of *AtSAHH1* (AT4G13940). An overnight pre-culture was 100-fold diluted in 500 mL of LB-Lennox media (1% tryptone (w/v), 0.5% (w/v) yeast extract, 0.5% (w/v) NaCl adjusted to pH 7 with NaOH) supplemented with 100 μg mL^{-1} ampicillin in a 2-L Erlenmeyer baffled flask. The culture was incubated to an OD_{600} of 0.6 (37°C, 180 rpm shaking rate), induced with 1 mM isopropyl β-D-1-thiogalactopyranoside (IPTG), and further cultivated at 16°C overnight. Cells were harvest by centrifugation (4,000*g*, 4°C, 15 min), flash frozen, and stored at -80°C until purification. For lysis, the bacteria pellet was resuspended in ice-cold lysis buffer (50 mM Tris-HCl pH 8.0, 300 mM NaCl, 20 mM imidazole, 0.5% (v/v) Triton® X-100, 1 mM β-mercaptoethanol; about 2.5 mL x g cell weight) and cells were lysed by three freeze-thaw cycles (-80°C freezer and 30°C water bath). Then, the lysate was supplemented with 1 mg mL^{-1} lysozyme and incubated on ice with gentle shaking for 30 min. After sonification, cellular debris were removed by centrifugation (18,000*g*, 20 min, 4°C) and the soluble faction was subjected to affinity chromatography using Ni-NTA agarose (Qiagen) and Econo-Pac® columns (Bio-Rad). The lysate was applied onto the column, equilibrated with lysis buffer, and washed with 10 CV of buffer A (50 mM Tris-HCl pH 8.0, 300 mM NaCl, 20 mM imidazole, 1 mM β-mercaptoethanol), 10 CV with buffer A supplemented with 1 M NaCl, and again with 10 CV of buffer A. Adsorbed proteins were eluted with buffer B (50 mM Tris-HCl pH 8.0, 300 mM NaCl, 300 mM imidazole, 20% (v/v) glycerol, 1 mM β-mercaptoethanol). The eluates were frozen in liquid nitrogen and stored at -80°C. Based on SDS-PAGE gels, fractions containing recombinant *At*SAHH1 protein were pooled together and rebuffered using Zeba™ Spin columns (Thermo Scientific) equilibrated with 50 mM Tris-HCl pH 8.0 before use. The protein content was determined by Bradford [1] using the Bio-Rad Protein Assay according to the manufacturer´s microplate protocol with BSA as standard.

SAHH activity assay

The activity of SAHH was determined in the SAH hydrolytic direction in the presence of excess adenosine deaminase (Ado). SAH is hydrolyzed to Hcys and adenine, which is subsequently converted into inosine and ammonia by Ado. This deamination is associated with the decrease in absorbance at 265 nm [2]. The reaction mixture contained 50 mM potassium-phosphate buffer pH 7.2, 1 mM EDTA, 0.75 U adenosine deaminase, and 2 μg purified recombinant protein in a final volume of 500 μl. After the addition of SAH to a final concentration of 100 μM, the decrease of absorbance at 265 nm at 10 s intervals for 300 s at RT in a *Ultrospec™ 3100 pro* UV/Visible spectrophotometer (Amersham Biosciences) using quartz suprasil® Hellma® Precission cells cuvettes (Hellma Analytics) was monitored. The reference sample contained water instead of SAH. The product concentrations were calculated from the slope of ΔA and $\varepsilon = 8.1\ mM^{-1}\ cm^{-1}$ (Ref [3]). The effect of GSNO or N-ethylmaleimide (NEM) on SAHH activity was analyzed by incubation of recombinant *At*SAHH1 (in 50 mM Tris-HCl pH 8) with these compounds for 30 min at RT in the dark. Thereafter, samples were desalted using Zeba™ Spin columns equilibrated with 50 mM Tris-HCl pH 8. To assess reversibility of GSNO-dependent inhibition of recombinant *At*SAHH1, 10 mM of the reducing agent dithiothreitol (DTT) was added 30 min following addition of GSNO, after which samples were incubated for 10 min prior to buffer exchange.

GSNOR activity assay

GSNOR activity in 4-week-old rosette leaves were determined as previously described [4]. Briefly, 0.5 g grinded plant material was extracted with 1 mL of extraction buffer (100 mM Tris–HCl pH 7.5, 0.1 mM EDTA, 0.2% (v/v) Triton® X-100, 10% (v/v) glycerol) on ice for 10 min with intermittent vortexing. The extracts were clarified by two centrifugation steps at 14,000*g* for 15 min at 4°C, and then, the protein concentration was determined according to Bradford [1] with BSA as standard. The reaction mixture

contained 20 mM Tris-HCl pH 8.0, 0.5 mM EDTA, 0.2 mM NADH, and 100 μg protein extract in a final volume of 1 mL. After the addition of GSNO to a final concentration of 500 μM, the oxidation of NADH was monitored by the decrease of absorbance at 340 nm (ε=6.22 mM^{-1} cm^{-1}) at 10 s intervals for 300 s at RT in a *Ultrospec™ 3100 pro* UV/Visible spectrophotometer (Amersham Biosciences) using quartz suprasil® Hellma® Precission cells cuvettes (Hellma Analytics). The reference sample contained water instead of GSNO. The activity was expressed as consumption of nmol NADH min^{-1} mg^{-1}.

Sodium dodecyl sulfate polyacrylamide gel electrophoresis

Sodium dodecyl sulfate polyacrylamide gel electrophoresis (SDS-PAGE) was performed according to Laemmli [5] as described by Sambrook and Russell [6]. Protein samples were diluted in 2x SDS sample buffer (4% (w/v) SDS, 20% (v/v) glycerol, 10% (v/v) 2-mercaptoethanol, 0.004% (w/v) bromophenol blue and 0.125 M Tris-HCl, pH 6.8.) and heated before electrophoresis for 5 min to 95°C. Proteins were separated by 10%, 12%, or 13.5% resolving gels and 5% SDS-PAGE stacking gels in SDS running buffer (25 mM Tris base, 192 mM glycine, 0.1% (w/v) SDS) on a Mini-PROTEAN® Tetra cell system (Bio-Rad). Running conditions were set to 200 V and 30 min. Proteins were visualized either by staining with *Coomassie*® Brilliant Blue G 250 or subjected to immunoblotting.

Coomassie® staining of SDS-PAGE gels

Gels were washed three times in deionized water for 2 min and then stained with Coomassie Brilliant Blue G 250 solution (0.1% Coomassie® G 250, 10% (v/v) glacial acetic acid, 45% (v/v) methanol) for 20 min while shaking. Destaining was allowed to proceed for several hours in deionized water or in destaining solution (10% (v/v) glacial acetic acid, 45% (v/v) methanol).

Immunoblotting

Electrophoretic separated proteins were blotted onto a Amersham™ Protran® 0.45 μM pore nitrocellulose membrane by semi-dry blotting using PerfectBlue™ Semi-Dry Electroblotter Sedec™ (VWR Peqlab) and Towbin buffer [7] (192 mM glycine, 25 mM Tris, 0.1% (w/v) SDS, 20% (v/v) methanol) at 2.5 mA cm^{-2} for 45 min at RT. Then blots were stained with Ponceau S to assess protein loading. After blocking in Tris-buffered saline buffer (TBS; 50 mM Tris-HCl pH 7.5, 150 mM NaCl) with 0.05% (v/v) Tween® (TBST) and 5% (v/v) BSA overnight, blots were incubated with primary antibody diluted in blocking buffer for 2 h at RT. Blots were washed thrice for 5 min in TBST. Then, the secondary antibodies in blocking buffer was added and the blot was further incubated for 1 h at RT. Thereafter, blots were washed thrice for 5 min in TBST and once with TBS. The bound secondary antibodies were detected by Western Lightning® Plus-ECL (PerkinElmer) chemiluminescence substrate and the luminograms were visualized using the Fusion FX7 imaging system (Vilber Lourmat). Signal intensities were measured using ImageJ software (National Institutes of Health) and *normalized* to *Ponceau S staining***.** The antibodies and dilutions used were: mouse monoclonal to H3K9me2 (1:1,000; ab1220 from Abcam), mouse monoclonal to biotin (1:5,000; A6561 Sigma), anti-rabbit IgG (1:2,500; W4011, Promega) and anti-mouse IgG (1:2,500; W4021, Promega), rabbit polyclonal to AtSAHH1 (1:10,000; Ref. [8]; kindly provided by Barbara Moffat).

Detection of S-nitrosated proteins

The biotin switch technique (BST; Ref. [9]) or the resin-assisted capture of S-nitrosothiols assay (RSNO-RAC; Ref. [10]) were used to analyze S-nitrosation of proteins. 10 μg of recombinant *At*SAHH1 in HEN buffer (100 mM Hepes-NaOH pH 7.7, 1 mM EDTA, 0.1 mM neocuproine) was treated with 50 μM, 250 μM, 500 μM GSNO or 500 μM GSH in a final volume of 100 μL for 30 min at RT in the dark with intermittent inverting of the tubes. Free thiols were blocked with 20 mM S-methylmethanethio-sulfonate (MMTS) in the presence of 2.5% (w/v) SDS at 300 rpm for 20 min at 50°C on a thermoshaker in the dark. After acetone precipitation to remove excess of GSNO and MMTS, the protein pellets were washed thrice with ice-cold acetone (10,000*g* for 10 min at 4°C), air-dried, and then resuspended in 32.5 μL of HENS buffer (HEN supplemented with 1% (w/v) SDS). The labeling reaction was started by the reduction of S-nitrosothiol

groups with sodium ascorbate (1 mM final concentration) to generate thiols and O-nitrosoascorbates via a transnitrosation reactions. The nascent reduced thiols, which were originally S-nitrosated, were then biotinylated by adding 1 mM biotin-HPDP. The labeling reaction (50 µL final volume) was allowed to proceed at 300 rpm for 1 h at RT using a thermoshaker in the dark. After acetone precipitation, the biotin-labeled proteins were resuspended in 45 µL non-reducing sample buffer (0.06 M Tris-HCl, pH 6.8, 10% (v/v) glycerol, 0.008% (w/v) bromophenol blue) and separated on a 10% SDS-PAGE gel without boiling prior to loading. Biotinylated proteins were detected with an anti-biotin antibody conjugated with alkaline phosphatase and 5-Bromo-4-chloro-3-indolyl phosphate/Nitro blue tetrazolium (BCIP/NBT) chromogenic visualization. *In planta,* S-nitrosation of *At*SAHH1 was investigated in 4-week-old *Arabidopsis* rosette leaves homogenized in HEN-T buffer (HEN supplemented with 0.2% (v/v) Triton® X-100 and protease inhibitor cocktail) in a ratio of 1:2. The extract was centrifuged twice at 18,000*g* for 20 min at 4°C. After desalting using Zeba™ Spin columns equilibrated with HEN buffer, the protein content was assayed by Bradford [1] using the Bio-Rad Protein Assay according to the manufacturer´s microplate protocol with BSA as standard. For *in vitro* S-nitrosation, extracts (1.5 mg; 1.13 µg μl^{-1}) were incubated with 0.5 mM GSNO or water as mock for 30 min at RT in the dark with frequent inverting. Next, free cysteine thiols were blocked by S-methylthiolation with 20 mM MMTS in the presence of 2.5% SDS at 300 rpm for 20 min at 50°C on a thermoshaker in the dark in a final volume of 1.5 mL. Excess of GSNO and MMTS was removed by precipitation with two volumes of ice-cold acetone for 20 min at -20°C. After centrifugation (10,000*g* for 10 min at 4°C), the protein pellet was washed three times with 70% (v/v) acetone, air-dried, and resuspended in HENS buffer (1 mL per mg protein). Afterwards 30 µL of the protein suspension were taken for analysis of total protein input. Next, 16 mg thiopropyl sepharose 6B per sample (equal to 50 µL bed volume) was equilibrated in excess HEN buffer for 15 min, washed thrice with HEN buffer by centrifugation (1,000*g* for 1 min) and resuspended by the addition of 150 µL HEN buffer. The protein suspension was added to 200 µL slurry (equal to 50 µL bed volume) in the presence of 50 mM ascorbate (prepared in HEN buffer) to a final volume of 2.3 mL. Thus, nascent SNO-proteins bind after their reduction to thiols with ascorbate to the thiol-reactive resin. Ascorbate was omitted for negative controls. Protein capture was allowed to proceed for 3 h at RT in the dark using an overhead shaker. To remove non-bond proteins, the resin was washed four times with 3 mL of HENS buffer and two times with 2 mL of HENS/10 buffer (HENS diluted 1:10). Centrifugation steps to collect beads were performed at 500*g* for 1 min. Originally S-nitrosated proteins were eluted with one bed volume of HENS/10 supplemented with 100 mM β-mercaptoethanol for 20 min at 22°C on a thermo-shaker (1500 rpm), separated on a 13.5% SDS-PAGE, and analyzed by immunoblotting with an anti-*At*SAHH1 antibody.

GUS activity staining

GUS activity was detected by histochemical staining according to Ref. [11] with modifications. *A. thaliana* line L5 harboring multiple copies of a 35Spro::*GUS* marker gene (*TS–GUS*; Morel et al. 2000) plantlets were immersed in fixing solution (50 mM sodium phosphate buffer pH 7.0, 0.05% (v/v) Triton® X-100, 0.5% (v/v) formaldehyde), vacuum infiltrated, and washed thrice with 50 mM sodium phosphate buffer pH 7.0. Then the plantlets were immersed into GUS staining solution (50 mM sodium phosphate buffer pH 7.0, 0.1% Triton® X-100, 1 mM potassium ferrocyanide, 1 mM potassium ferricyanide, 1 mM 5-bromo-4-chloro-3-indolyl-D-glucuronic acid) and incubated at 37°C overnight. Plantlets were washed once with 70% (v/v) ethanol and incubated in 70% (v/v) ethanol at 80°C to remove chlorophyll for 10 min.

Quantification of S-nitrosothiols

The S-nitrosothiol level in 4-week-old rosette leaves were determined by triiodide-dependent ozone-based chemiluminescence using the Nitric Oxide Analyzer Sievers® 280i from GE Healthcare. This method is based on the reduction of nitrite and RSNO by triiodide to NO, which reacts with ozone to form the excited state of nitrogen dioxide (NO_2) and O_2. Upon decay to its ground state, NO_2* emits a photon which is detected by a photomultiplier [13]. The intensity of emitted light is directly proportional to the amount of NO. In short, rosette leave extracts were prepared by homogenization of 500 mg ground plant material in

two volumes of 1x phosphate buffered saline (PBS; 137 mM NaCl, 2.7 mM KCl, 4.3 mM Na_2HPO_4, 1.47 mM KH_2PO_4, pH 7.4) and incubation on ice for 10 min with intermittent vortexing. After centrifugation at 18,000*g* for 15 min at 4°C, the supernatant was used for further analysis. For RSNO measurements, extracts were pre-treated with 5% sulfanilamide (w/v, in 1 M HCl) at a ratio of 9:1 (extract : sulfanilamide) to scavenge nitrite and 200 µL were injected into the reaction vessel containing acidic triiodide as reducing agent (28.5 mM I_2, 66.9 mM KI, 77.7% glacial acetic acid; 30°C). The peak area integration and quantification of RSNO content were performed with Sievers® NOA Analysis™ software (GE Healthcare) using nitrite standards and normalized to the protein content assayed according to Bradford [1].

References

1. Bradford, M.M. A rapid and sensitive method for the quantitation of microgram quantities of protein utilizing the principle of protein-dye binding. *Anal. Biochem.* **1976**, *72*, 248–254, doi:https://doi.org/10.1016/0003-2697(76)90527-3.
2. Fujioka, M.; Takata, Y. S-Adenosylhomocysteine hydrolase from rat liver. Purification and some properties. *J. Biol. Chem.* **1981**, *256*, 1631–1635, doi:10.1139/o82-016.
3. Takata, Y.; Yamada, T.; Huang, Y.; Komoto, J.; Gomi, T.; Ogawa, H.; Fujioka, M.; Takusagawa, F. Catalytic mechanism of S-adenosylhomocysteine hydrolase. Site-directed mutagenesis of Asp-130, Lys-185, Asp-189, and Asn-190. *J Biol Chem* **2002**, *277*, 22670–22676, doi:10.1074/jbc.M201116200.
4. Sakamoto, A.; Ueda, M.; Morikawa, H. Arabidopsis glutathione-dependent formaldehyde dehydrogenase is an S-nitrosoglutathione reductase. *FEBS Lett.* **2002**, *515*, 20–24, doi:10.1016/S0014-5793(02)02414-6.
5. Laemmli, U.K. Cleavage of Structural Proteins during the Assembly of the Head of Bacteriophage T4. *Nature* **1970**, *227*, 680–685, doi:10.1046/j.1365-313x.1998.00343.x.
6. Sambrook, J.; Russell, D.W. *Molecular cloning: a laboratory manual*; 3rd ed.; Cold Spring Harbor Laboratory Press: New York, 2001; ISBN 0879695773 (paperback).
7. Towbin, H.; Staehelin, T.; Gordon, J. Electrophoretic transfer of proteins from polyacrylamide gels to nitrocellulose sheets: procedure and some applications. *Proc. Natl. Acad. Sci. U. S. A.* **1979**, *76*, 4350–4, doi:10.1002/bies.950190612.
8. Pereira, L.A.; Todorova, M.; Cai, X.; Makaroff, C.A.; Emery, R.J.; Moffatt, B.A. Methyl recycling activities are co-ordinately regulated during plant development. *J Exp Bot* **2007**, *58*, 1083–1098, doi:10.1093/jxb/erl275.
9. Jaffrey, S.R.; Snyder, S.H. The Biotin Switch Method for the Detection of S-Nitrosylated Proteins. *Sci. Signal.* **2001**, *2001*, pl1–pl1, doi:10.1126/stke.2001.86.pl1.
10. Forrester, M.T.; Thompson, J.W.; Foster, M.W.; Nogueira, L.; Moseley, M.A.; Stamler, J.S. Proteomic analysis of S-nitrosylation and denitrosylation by resin-assisted capture. *Nat. Biotechnol.* **2009**, *27*, 557–559, doi:10.1038/nbt.1545.
11. Jefferson, R.A.; Kavanagh, T.A.; Bevan, M.W. GUS fusions: beta-glucuronidase as a sensitive and versatile gene fusion marker in higher plants. *EMBO J.* **1987**, *6*, 3901–7, doi:10.1073/pnas.1411926112.
12. Morel, J.-B.; Mourrain, P.; Béclin, C.; Vaucheret, H. DNA methylation and chromatin structure affect transcriptional and post-transcriptional transgene silencing in Arabidopsis. *Curr. Biol.* **2000**, *10*, 1591–1594, doi:http://dx.doi.org/10.1016/S0960-9822(00)00862-9.
13. Piknova, B.; Schechter, A.N. Measurement of Nitrite in Blood Samples Using the Ferricyanide-Based Hemoglobin Oxidation Assay. *Methods Mol. Biol.* **2011**, *704*, 39–56, doi:10.1007/978-1-61737-964-2_4.
